# LRMP Associates With Immune Infiltrates and Acts as a Prognostic Biomarker in Lung Adenocarcinoma

**DOI:** 10.3389/fmolb.2021.711928

**Published:** 2021-11-26

**Authors:** Xin Jin, Liwei Chen, Ning Zhou, Hong Ni, Lingling Zu, Jinling He, Lingqi Yang, Yifan Zhu, Xiaoyue Sun, Xiaojiang Li, Song Xu

**Affiliations:** ^1^ Department of Lung Cancer Surgery, Tianjin Medical University General Hospital, Tianjin, China; ^2^ Tianjin Key Laboratory of Lung Cancer Metastasis and Tumor Microenvironment, Lung Cancer Institute, Tianjin Medical University General Hospital, Tianjin, China; ^3^ Department of Oncology, First Teaching Hospital of Tianjin University of Traditional Chinese Medicine, Tianjin, China

**Keywords:** LRMP, lung adenocarcinoma, prognosis, immune infiltration, immune checkpoints (ICP)

## Abstract

**Background:** Lymphoid-restricted membrane protein (LRMP) is an endoplasmic reticulum-associated protein that is expressed in a developmentally regulated manner in both B and T cell lineages. However, the role of LRMP in the growth, prognosis and immune infiltration in lung adenocarcinoma (LUAD) remains unclear.

**Method:** The expression levels of *LRMP* mRNA in tumor and normal tissues were analyzed using Tumor Immune Estimation Resource 2.0 (TIMER 2.0) and Gene Expression Profiling Interactive Analysis 2 (GEPIA 2). LRMP protein expression was examined using the Human Protein Atlas. *In vitro* experiments, including qRT-PCR Western blot and immunohistochemistry staining were also performed to investigate LRMP expression. GEPIA2 and Kaplan-Meier plotter databases were used to analyze the clinical prognostic significance of LRMP. To further confirm the underlying function of LRMP, the data were analyzed using gene set enrichment analysis. Moreover, we also constructed plasmids to overexpress LRMP and explored the effect of LRMP in A549 cell line. Additionally, Tumor Immune single-cell Hub was used to investigate the distribution of LRMP in the LUAD immune microenvironment; TIMER and CIBERSORT were used to investigate the relationships among *LRMP*, *LRMP* co-expressed genes, and tumor-infiltrating immune cells; Finally, the correlations between LRMP and immune checkpoints were analyzed using TIMER 2.0.

**Results:** The expression of *LRMP* was significantly lower in LUAD tissues and cell lines. High *LRMP* expression is associated with a better prognosis in patients with LUAD. *In vitro* experimental studies demonstrated that overexpression of LRMP could decrease the proliferation, migration and invasion in A549 cells, and downregulated multiple oncogenic signaling pathways, including p-STAT3, p-PI3K-p-AKT, p-MEK and EMT pathways. GSEA results showed that immuno-related and cell adhesion pathways were enriched in samples with high LRMP expression. LRMP and its co-expressed genes were positively correlated with various tumor-infiltrating immune cells and their markers. Additionally, LRMP positively correlated with immune checkpoints.

**Conclusions:** Our data suggest that LRMP may act as a tumor suppressor gene and indicates a better prognosis. Moreover, LRMP is associated with immune infiltrates which may be involved in immunotherapy response in LUAD. Further studies are needed to validate these findings.

## Introduction

Lung cancer is the second most common type of cancer in 2020 and is the leading cause of cancer-related deaths, accounting for approximately one in 10 cancers diagnosed and one in five deaths (Sung et al., 2021). Eighty-four percent of lung cancers are non-small cell lung cancers (NSCLCs), and only 20–30% of newly diagnosed lung tumors can be treated with radical surgery (Noone et al., 2018). Immunotherapy, such as programmed death-1 (PD-1), programmed death ligand-1 (PD-L1), and cytotoxic T lymphocyte-associated antigen 4 inhibitors, have shown promising results in melanoma, renal cancer, NSCLC, and other tumors (Garon et al., 2015; Larkin et al., 2015; Motzer et al., 2015). Although immune checkpoint inhibitors have shown significant clinical efficacy and long-lasting responses in patients with NSCLC, the overall response rate of anti-PD-1/PD-L1 therapy is only 20–30% (Borghaei et al., 2015; Herbst et al., 2019). Effective predictive biomarkers are specific for the selection of patients for immunotherapy. Therefore, it is urgent to identify biomarkers related to the immune interaction with NSCLC, as well as biomarkers favorable for immunotherapy, including tumor cell-associated biomarkers, TME-related biomarkers, liquid biopsy-related biomarkers, and host-related markers (Bai et al., 2020).

*LRMP*, also known as *JAW1*, first reported in 1994, encodes 539 amino acid proteins located in the endoplasmic reticulum of lymphocytes and plays a role in lymphoid development (Behrens et al., 1994). Specifically, LRMP may be involved in the intracellular transport of developmentally regulated antigen receptors in lymphocytes. LRMP proteins can effectively transport COOH-terminal antigenic peptides to MHC class I molecules in an antigen-processing-independent pattern (Snyder et al., 1997). Many studies have revealed that coding variations of *LRMP* in mice are closely associated with changes in strain susceptibility to lung tumorigenesis, whether spontaneous or chemically induced (Manenti et al., 2004; Manenti and Dragani, 2005), while coding or non-coding SNPs of human homologous genes are not significantly associated with lung cancer risk. However, patients with tumor onset ages ≤65 years with Leu Variation (V141L) in *LRMP* had higher mortality (Manenti et al., 2006). Additionally, previous studies found that classical Hodgkin’s disease and myeloma lack LRMP; however, many chronic lymphocytic leukemias are positive for LRMP (Tedoldi et al., 2006). Moreover, LRMP was identified as a favorable prognostic predictor in diffuse large B-cell lymphoma (Zhang et al., 2007). Recent studies have shown that *LRMP* is expressed at low or moderate levels in other human tissues such as the brain, lungs, pancreas, bladder, ovary, and skin, and is also assumed to be a component of the linker of the nucleus and cytoskeletons (LINC) complex, which is involved in maintaining the shape of the nucleus (Kozono et al., 2018). The LINC complex mediates cell polarity and proliferation after activation (Obino et al., 2016; Donahue et al., 2017). However, the underlying mechanism of LRMP in LUAD progression and immunology remains unclear.

In the present study, we aimed to investigate the expression of *LRMP* in lung cancer and its correlation with immune infiltrates and survival in lung cancer using public databases and bioinformatics web tools. These findings will help to identify potential biomarkers for the prognosis of lung cancer and contribute to individualized immunotherapy.

## Materials and Methods

### TCGA Database

The Cancer Genome Atlas (TCGA) (https://www.cancer.gov/tcga) is a publicly funded project aimed at cataloging and discovering major oncogenic genomic alterations in a large cohort of more than 30 human tumors through large-scale genome sequencing. TCGA includes RNA sequencing, miRNA sequencing, DNA sequencing, DNA methylation sequencing, and clinical information (Tomczak et al., 2015). Transcriptome and clinical data of LUAD patients were downloaded from TCGA, and the expression matrix and clinical information of 513 patients were obtained through collation.

### GEPIA 2 Database Analysis

GEPIA2 (http://gepia2.cancer-pku.cn/) (Tang et al., 2019) is an updated version of gene expression profiling interactive analysis (GEPIA) (Tang et al., 2017) that was used to analyze RNA sequencing expression data from 9,736 tumors and 8,587 normal samples from the TCGA and GTEX programs. Tumor/normal differential expression analysis, patient survival analysis, similar gene detection, correlation analysis, and dimensionality reduction analysis were performed. GEPIA2 was used to analyze *LRMP* expression differences matching TCGA data and GTEX data, and to analyze the prognostic significance of LRMP in patients with LUAD.

### The Human Protein Atlas

The Human Protein Atlas (HPA) (https://www.proteinatlas.org/) was initiated in 2003 to map all human proteins in cells, tissues, and organs using a variety of omics techniques, including antibody-based imaging, mass spectrometer-based proteomics, transcriptomics, and systems biology. HPA consists of tissue, single cell type, pathology, blood, brain, and cell atlases. We searched “LRMP” in the pathological atlas of HPA (“lung cancer” as the cancer type). Then we checked the details of each image and selected the representative images of cancer and adjacent samples in LUAD.

### Kaplan-Meier Plotter Database Analysis

The Kaplan Meier plotter (http://kmplot.com/analysis/) (Nagy et al., 2018) was used to evaluate the effect of 54 K genes (mRNA, miRNA, and protein) on survival (*n* = 1,440) of 21 cancer types, including breast cancer (*n* = 7,830), ovarian cancer (*n* = 2,190), lung cancer (*n* = 3,452), and gastric cancer. The database sources included GEO, EGA, and TCGA. The correlation between *LRMP* expression and survival [overall survival (OS), first progression [FP], post-progression survival (PPS)] in patients with LUAD was analyzed using the Kaplan-Meier Plotter (Győrffy et al., 2013). Hazard ratios (HRs) with 95% confidence intervals and log-rank *p* values were calculated.

### TISCH Database Analysis

Tumor Immune Single-cell Hub (TISCH) (http://tisch.comp-genomics.org) integrates nearly two million single-cell transcriptome data from 76 high-quality datasets for 27 cancers, aiming to characterize the tumor microenvironment at a single-cell resolution (Sun et al., 2021a). TISCH visualizes gene expression across multiple data sets at the single-cell or cluster level, enabling comparisons between different patients, treatment and response groups, tissue origins, cell types, and even different cancer types. We firstly selected eight NSCLC datasets from TISCH. TISCH automatically analyzed the composition of tumor immune microenvironment in these datasets. We then searched GEO database and Pubmed and selected two datasets (EMTAB6149 and GSE131907) which only included LUAD from the above eight datasets. We further searched “LRMP” in TISCH which automatically displayed the distribution of LRMP.

### TIMER and TIMER 2.0 Database Analysis

Tumor Immune Estimation Resource (TIMER) (https://cistrome.shinyapps.io/timer/) is a comprehensive analytic web tool that provides six modules to explore immune infiltrates and different factors including gene expression, clinical outcomes, somatic copy number alterations, and somatic mutations (Li et al., 2017). The TIMER database integrates 10,897 samples across 32 cancer types from the TCGA. A previously published deconvolution statistical method was applied to estimate the abundance of immune infiltrates through gene expression profiling (Li et al., 2016). TIMER 2.0 (http://timer.cistrome.org/) is an updated web server of TIMER using six different computational methods to infer immune cell infiltration (Li et al., 2020). After submitting “LRMP” in the Diff Exp Module of TIMER, TIMER automatically generates the expression level of LRMP in different cancer types. After submitting “LRMP” and selecting the cancer type as LUAD in the Gene module, TIMER automatically generates the relationship images between LRMP and immune cells. Similarly, the relationship between LRMP co-expressed genes and immune cells was analyzed as well. Gene expression levels against the degree of tumor purity are shown in the left-most panel (Aran et al., 2015). Additionally, the association between *LRMP* expression and markers of tumor-infiltrating immune cells was explored using the TIMER correlation module. In the correlation module of TIMER, LUAD was selected as the cancer types, and the expression level of LRMP was X-axis and the gene of interest was Y-axis. Correlation curves were generated automatically after submission. The molecular markers were referenced from previous studies (Sousa and Määttä, 2016; Danaher et al., 2017; Siemers et al., 2017). The relationship between LRMP and co-expressed genes was also verified in TIMER using the same method. TIMER 2.0, was used to analyze the relationship between *LRMP* expression and immune checkpoints in Gene_Corr module. Gene expression levels were determined by log2 RSEM.

### Gene Set Enrichment Analysis

Transcriptome data of 513 LUAD patients were downloaded from TCGA and divided into groups with high or low LRMP expression based on the median value of *LRMP* expression. GSEA detected pathways enriched by the top-ranked genes in both groups. In each analysis, the gene set permutations were set to 1,000. The normalized enrichment score (NES), nominal (NOM) *p*-value, false discovery rate (FDR), and family-wise error rate (FWER) were used to appraise the enriched pathways.

### Analysis of Genes Co-expressed With LRMP

To further study the molecular mechanism related to LRMP, we identified the genes co-expressed with *LRMP* using the cBioPortal database (https://www.cbioportal.org/) (Cerami et al., 2012) and screened the proteins interacting with LRMP. We selected six highly significant genes correlated with LRMP for further analysis, and the correlation between them was verified using the TIMER database. The expression of these genes co-expressed with *LRMP* in LUAD was analyzed using the TIMER database. The relationships between these genes and LUAD stages were analyzed using the TISIDB database (Ru et al., 2019). The prognostic significance of the genes co-expressed with *LRMP* in LUAD was explored using GEPIA 2.

### LUAD Tissues and Cell Culture

A total of 5 paired LUAD tissues and their corresponding adjacent normal lung tissues were obtained from LUAD patients who underwent resection without preoperative therapy at Tianjin Medical University General Hospital (Tianjin, China). This study was approved from Medical Ethics Committee of our hospital (Ethical NO. IRB2021-wz-152). All patients provided a written informed consent. The LUAD cell lines (H1792, H1975, H23, H1299 and A549) and the human normal lung epithelial cell line BEAS-2B were obtained from the American Type Culture Collection (ATCC)and cultured in RPMI 1640 with 10% fetal bovine serum.

### Quantitative Reverse Transcription Polymerase Chain Reaction

Total RNA was extracted from cell lines with a TRIzol reagent (Invitrogen). cDNA synthesis was carried out using Prime Script RT Master Mix (TaKaRa, Dalian, China), while reverse transcription-polymerase chain reaction (PCR) was performed using TB Green Premix Ex Taq (TaKaRa) on the 7900HT Fast Real‐Time PCR System (Applied Biosystems, Foster City, CA, USA; Thermo Fisher Scientific). QRT-PCR was performed to detect mRNA levels of LRMP, IL16, KLHL6, EVI2B, SASH3, ARHGAP25, IKZF1, CD274 and GAPDH. GAPDH was used as the endogenous control. Each gene was repeated three times. The primer sequences were listed in supplementary materials.

### Western Blotting

Cell lines were collected and lysed in RIPA lysis buffer with PMSF (Beyotime, Shanghai, China). Protein samples were separated by sodium dodecyl sulphate–polyacrylamide gel electrophoresis and transferred in the polyvinylidene difluoride membranes (Millipore). Then, 5% BSA was used to block the membranes for 2 h. Subsequently, the membranes were incubated with primary antibodies at 4°C overnight and incubated with the corresponding horseradish peroxidaseconjugated secondary antibody. Each band was visualized by enhanced chemiluminescence reagents (Yeasen, Shanghai, China) The western blotting was performed to detect the protein expression level of LRMP, p-STAT3, p-PI3K, p-AKT, p-MEK, E-cadherin, N-cadherin, slug and GAPDH. GAPDH was used as the endogenous control. The information of antibodies was listed in supplementary materials.

### Immunohistochemistry

The sections of 5 pairs of LUAD and paired normal lung tissues were placed in an oven at 56°C for 1 h to melt the paraffin and prevent the tissues from shedding, prior to dewaxing. The paraffin was removed with xylene and alcohol and then the sections were placed in sodium citrate buffer (pH 6.0) for repair. The sections were repaired in a pressure cooker for 15 min, cooled to room temperature, and then rinsed in phosphate buffered saline (PBS) for 3 times. The sections were placed in 3% hydrogen peroxide solution and incubated for 15 min at room temperature to inactivate endogenous peroxidase in the tissues. The sections were incubated with anti-LRMP primary antibody, diluted in the ratio of 1:100 (Abcam, ab202418, US) at 4°C overnight. Next, the sections were incubated with secondary antibody for 1 h, and then washed 3 times in PBS, for 5 min each. Thereafter, diaminobenzidine tetrachloride (DAB) was added and then rinsed after satisfactory color development. We then stained the sections with hematoxylin for 25 s and rinsed for 20 min. Finally, we covered the sections with neutral balsam and cover slips. And a pathologist was consulted to ensure the typicality of selected tissues.

### Overexpression Vector and Transfection

A549 cells were grown in RPMI-1640 containing 10% fetal bovine serum at 37°C in a 5% CO_2_ incubator. 5 × 10^5^cells were seeded on each well of 6-well plates the day before transfection. The pCMV-vector and pCMV-LRMP vector were transfected into A549 cells by Lipofectamine 2000 according to the manufacturer’s instructions. After 48 h transfection, cells were harvested for total RNA, whole cell lysates extractionand functional exploration.

### Cell Counting Kit-8 Assays

In line with the manufacturers’ protocols, Cell Counting Kit-8 (CCK-8) (Dojindo, Japan) was used to analyze the proliferation of LUAD cells. Briefly, the LUAD cells (3000cells/200 μL) were seeded, and 20 μL of Cell Counting Kit-8 solution was added at the same time of each day. After incubating for 2 h in an incubator, the absorbance (450 nm) was measured.

### Migration and Invasion Assays

Transwell inserts (Corning Inc., Corning, NY, USA) with or without Matrigel (BD Biosciences, San Diego, CA, USA) were used to evaluate cell invasion and migration, respectively. For cell invasion assay, the upper chambers were coated with Matrigel. A total of 12 × 10^4^ cells were seeded into the upper chamber filled with 300 ml of serumfree medium. Then, the lower chambers were added with 600 ml of containing 10% fetal bovine serum. After 48 h of incubation, the invaded cells were fixed with 4% paraformaldehyde, stained with 1% crystal violet, and photographed under a microscope. Cell migration assay was carried out in a similar manner without coating the upper chambers with Matrigel, and the results was observed after 24 h of incubation,3 × 10^4^ cells were seeded into the upper chamber.

### Statistical Analysis

R 4.0.3 software, ggplot2 and GraphPad Prism 9 were used for statistical analysis, and the significance of the two groups of samples was analyzed using the Wilcoxon test. The association between *LRMP* expression and clinical characteristic variables was analyzed using Pearson’s chi-square test. The Cox proportional risk regression model was used for the univariate and multivariate analyses. Statistical significance was set at *p* < 0.05.

## Results

### Decreased Expression of LRMP in LUAD

**FIGURE 1:**
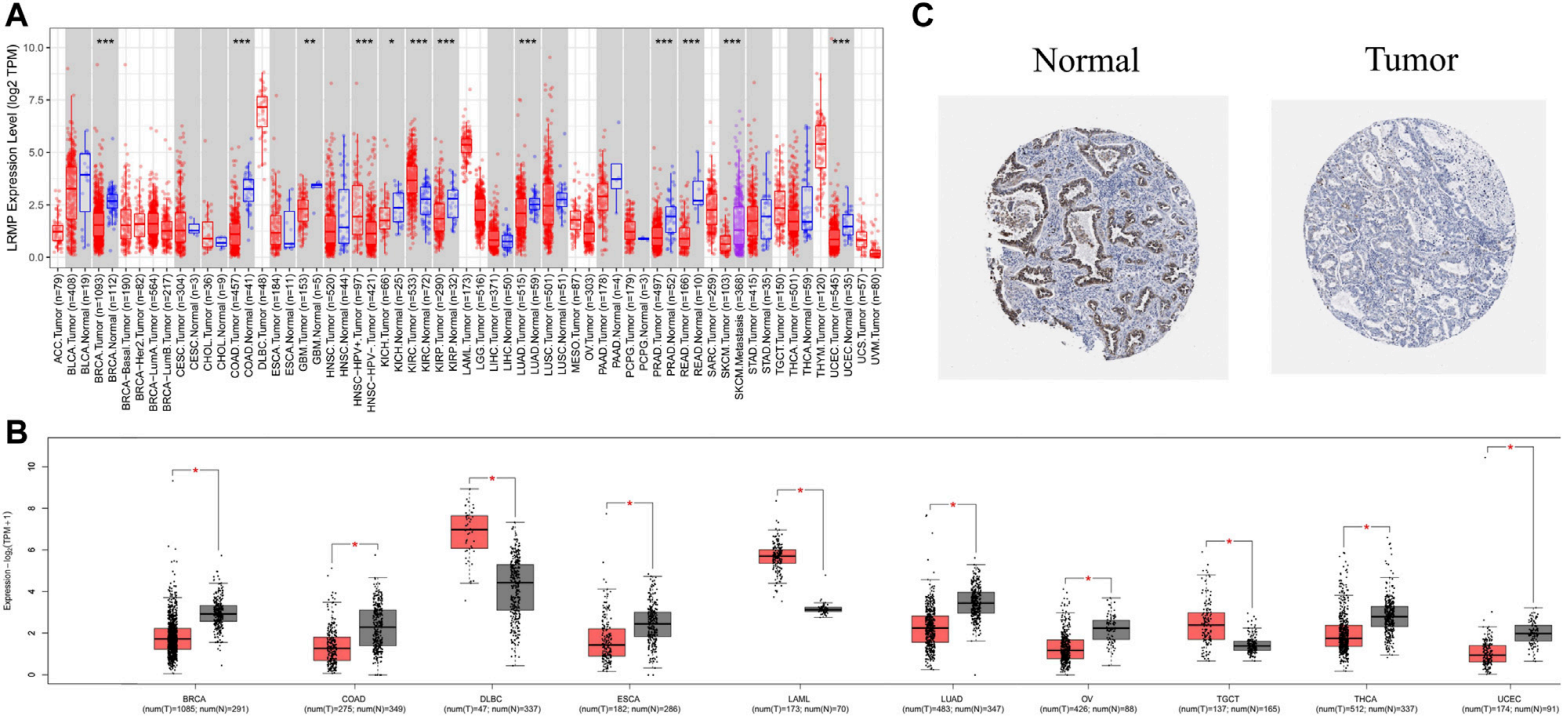
The expression level of lymphoid-restricted membrane protein (LRMP) in different human cancers. **(A)** Increased or decreased expression of *LRMP* in cancers compared with adjacent normal tissue in Tumor Immune Estimation Resource (TIMER) database. **(B)** *LRMP* expression level in different cancers compared with GTEX data in GEPIA 2. **(C)** LRMP protein expression in normal tissue and tumor tissue of patients with LUAD.

To investigate the difference in LRMP expression levels between tumor and normal tissues, we analyzed RNA-seq data using TIMER 2.0, and GEPIA 2. TIMER 2.0, matched TCGA tumor tissue with normal tissue, and GEPIA 2, matched TCGA tumor tissue with GTEX data. TIMER 2.0 results (Figure 1A) showed that mRNA levels of *LRMP* were significantly lower in LUAD tissues than in normal tissues. We also found that *LRMP* was low in most solid cancers, including breast invasive carcinoma, colon adenocarcinoma, glioblastoma multiforme, kidney chromophobe, kidney renal papillary cell carcinoma, prostate adenocarcinoma, rectum adenocarcinoma, and uterine corpus endometrial carcinoma. The lower expression of *LRMP* in LUAD was also confirmed by GEPIA 2 (Figure 1B). Moreover, LUAD patients showed lower LRMP protein expression compared to normal lung tissues in The HPA (Figure 1C). These data suggest that LUAD patients had decreased *LRMP* expression.

**FIGURE 2:**
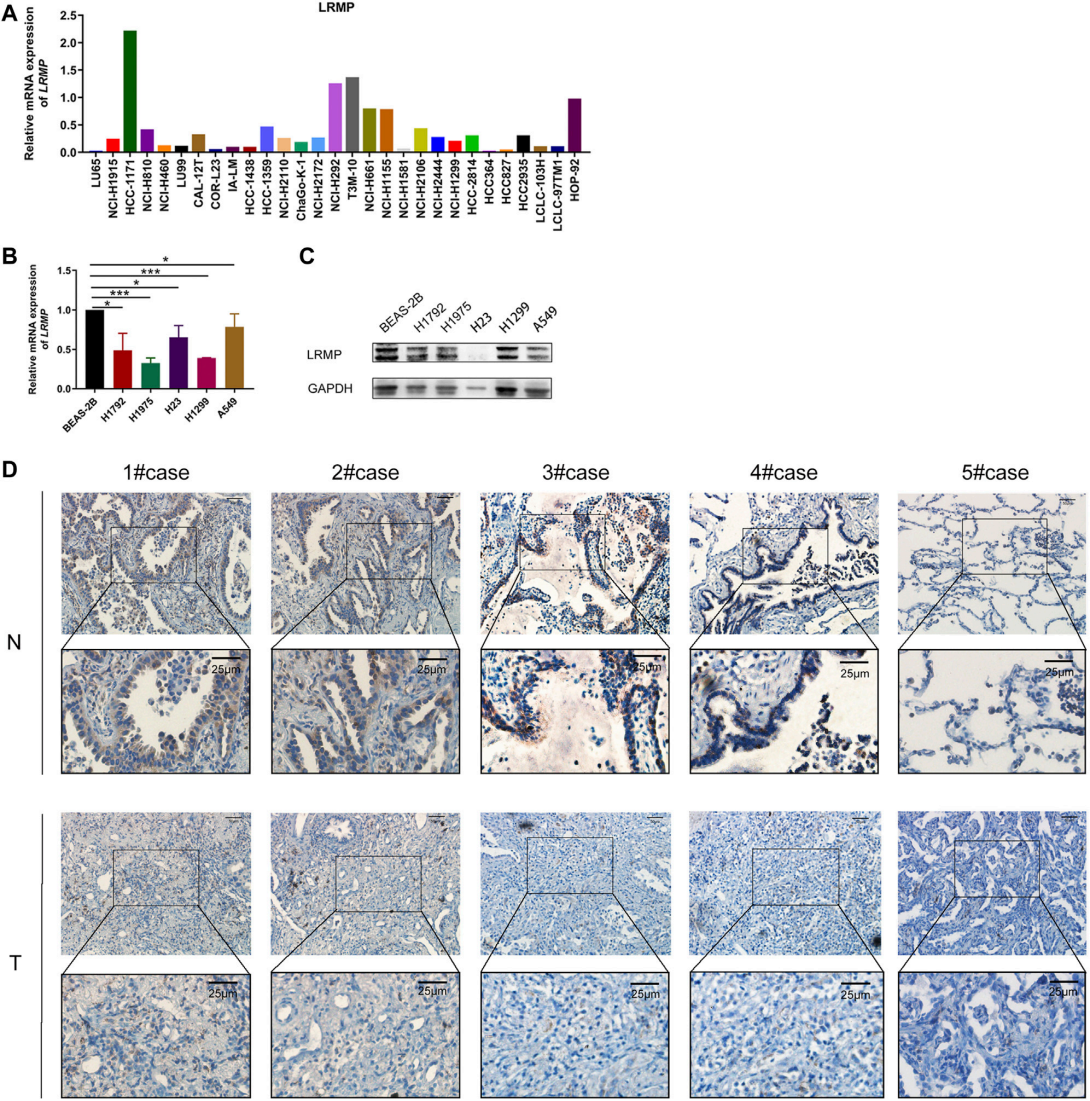
The expression level of lymphoid-restricted membrane protein (LRMP) in cell lines and LUAD tissue. **(A)** LRMP mRNA level in different cell lines according to CCLE. **(B–C)** mRNA and protein expression of LRMP in LUAD cell lines. **(D)** Representative IHC figures of LUAD tissues and paired normal tissue stained for LRMP protein.

To further verify the analysis, we explored the mRNA expression levels of LRMP in the CCLE database (Figure 2A). Furthermore, we compared the mRNA and protein levels of LRMP in lung normal epithelial cell line and LUAD cell lines by qRT-PCR and Western blotting (Figures 2B–C), and confirmed that LRMP was down-regulated in both mRNA and protein levels in LUAD cell lines. In addition, immunohistochemical staining also demonstrated that the LRMP expression of LUAD was decreased compared to the corresponding adjacent normal lung tissues. The representative images are shown in Figure 2D. In addition, we found that there was a significantly decreased trend of LRMP expression from stage I to stage IV in LUAD (*p* = 9.93e-05) (Supplementary Figure S1A). We also performed subgroup analysis and found there was no significant difference of LRMP expression between EGFR/ALK mutation and wild type LUAD patients (Supplementary Figure S1B,C).

### Relationship Between LRMP and Clinicopathological Variables in LUAD Patients

The association between *LRMP* expression and clinicopathological variables including vital status, age, sex, race, T stage, N stage, M stage, TNM stage, new tumor event type, and smoking history of LUAD patients was investigated using TCGA data. Based on median *LRMP* mRNA levels, 257 patients were assigned to the group (G1) with high *LRMP* expression and 256 patients to the group (G2) with low *LRMP* expression. As shown in Table 1, *LRMP* expression was significantly correlated with vital status (*p* = 0.001), age (*p* = 0.03), sex (*p* = 0.007), TNM stage (*p* = 0.02), and new tumor event type (*p* = 0.033). There was no significant correlation between *LRMP* expression and other clinicopathological characteristics, including race (*p* = 0.393), T stage (*p* = 0.088), N stage (*p* = 0.091), M stage (*p* = 0.07), and smoking history (*p* = 0.083).

**TABLE 1:** Relationship between LRMP expression and clinical variates in LUAD patients.

	Characteristic	G1	G2	*p* Value
status	alive	182	144	0.001
	dead	75	112	
age	mean (SD)	66.3 (9.9)	64.3 (10.1)	0.03
	Median (min, max)	67 (33, 88)	65 (40, 87)	
gender	female	154	122	0.007
	male	103	134	
race	Asia	2	5	0.393
	black	24	28	
	white	199	188	
	America Indian		1	
T stage	T1	38	28	0.088
	T1a	26	21	
	T1b	34	21	
	T2	78	89	
	T2a	40	42	
	T2b	16	11	
	T3	15	32	
	T4	8	11	
	Tx	2	1	
N stage	N0	178	152	0.091
	N1	38	57	
	N2	32	42	
	N3	1	1	
	Nx	7	4	
M stage	M0	159	185	0.07
	M1	7	11	
	M1a	1	1	
	M1b	3	2	
	Mx	84	56	
TNM stage	Ⅰ	1	4	0.02
	ⅠA	82	48	
	ⅠB	71	68	
	Ⅱ		1	
	ⅡA	22	28	
	ⅡB	29	41	
	ⅢA	33	40	
	ⅢB	3	8	
	Ⅳ	12	14	
new tumor event type	metastasis	26	35	0.033
	Metastasis:recurrence	4	4	
	primary	10	1	
	recurrence	25	23	
	metastasis:primary		1	
smoking	non-smoking	44	30	0.083
	smoking	203	222	

### Prognostic Value of LRMP in LUAD

**FIGURE 3:**
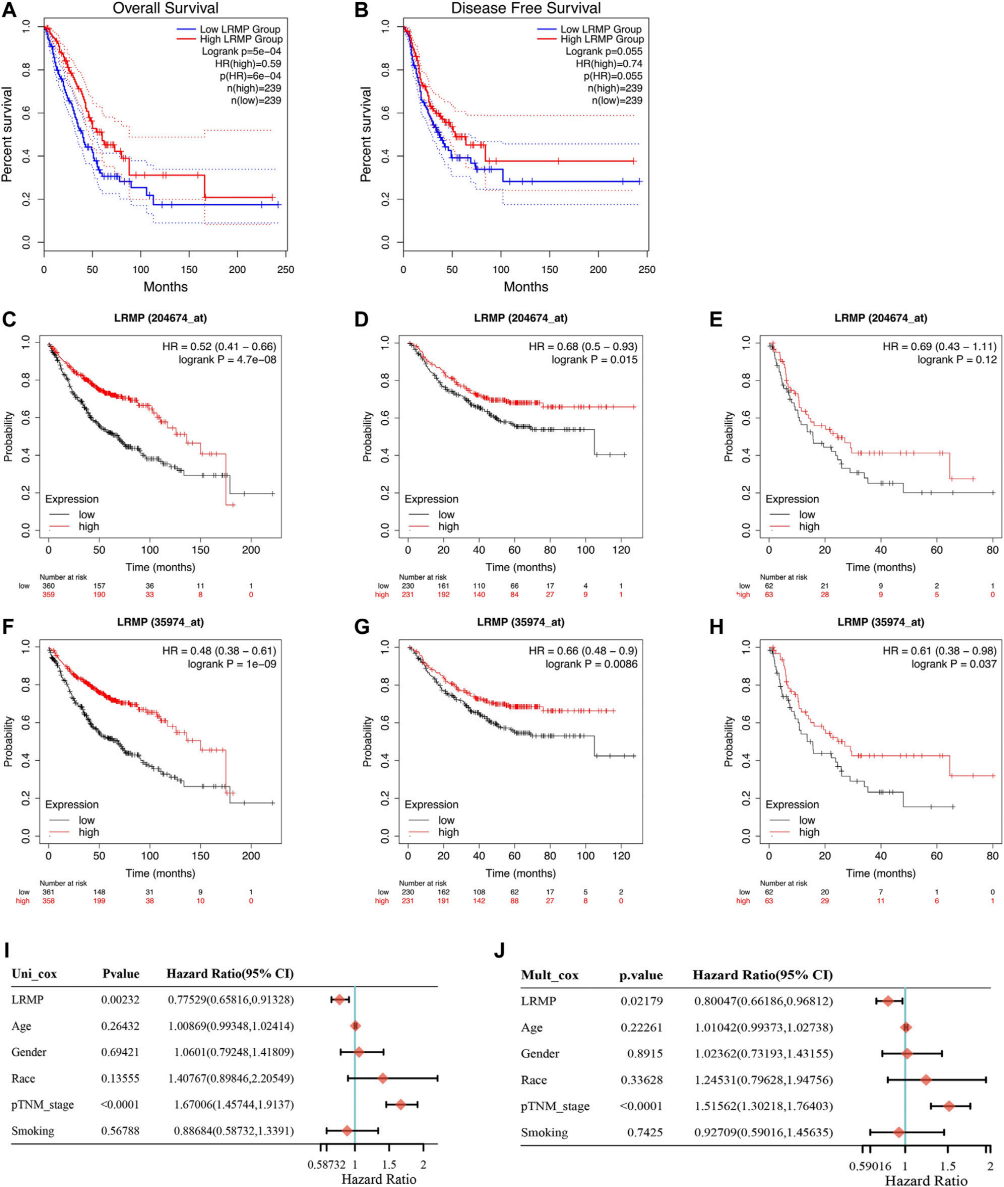
The prognostic value of lymphoid-restricted membrane protein (LRMP) in patients with lung adenocarcinoma (LUAD). **(A–B)** Overall survival and disease-free survival of patients with LRMP ^high^ and LRMP ^low^ LUAD analyzed in GEPIA 2. **(C–E)** Overall survival, progression-free survival, and post-progression survival of patients with LUAD in Kaplan-Meier database with Affy ID: 204674_at based on *LRMP* expression levels. **(F–H)** Overall survival, progression-free survival, and post-progression survival of patients with LUAD in Kaplan-Meier database with Affy ID: 35974_atbased on *LRMP* expression levels. **(I–J)** Cox analysis showing the hazard ratios (HRs) of different factors.

To investigate the prognostic value of LRMP in LUAD, we analyzed TCGA data using GEPIA 2 and the Kaplan-Meier plotter database. In the GEPIA 2 data (Figures 3A,B), the high expression of *LRMP* was significantly associated with longer OS (HR = 0.59, *p* = 6e−04); however, not with disease-free survival (HR = 0.74, *p* = 0.055). In the Kaplan-Meier plotter database, two probes were used to detect the LRMP. In the results of AFFY ID:204674_at (Figures 3C–E), high expression of *LRMP* was associated with better OS (HR = 0.52, *p* = 4.7e−08) and FP (HR = 0.68, *p* = 0.015); however, not with PPS (HR = 0.69, *p* = 0.12). In the results of AFFY ID: 35974_at (Figures 3F–H), high expression of LRMP was significantly associated with better OS (HR = 0.48, *p* = 1e−09), FP (HR = 0.66, *p* = 0.0086), and PPS (HR = 0.61, *p* = 0.037).

Furthermore, univariate and multivariate Cox survival analyses were used to demonstrate that *LRMP* expression was an independent predictor of favorable prognosis in LUAD (Figures 3I, J). The TNM stage (*p* < 0.0001) and *LRMP* expression (*p* = 0.00232) significantly affected the survival of patients with LUAD in univariate Cox survival analysis, and similar results were found in multivariate Cox survival analysis [TNM stage (*p* < 0.0001) and LRMP (*p* = 0.02179)]. Our results indicate that LRMP is a positive prognostic predictor and an independent prognostic biomarker.

### GSEA Identifies LRMP-Related Signaling Pathways in LUAD

**FIGURE 4:**
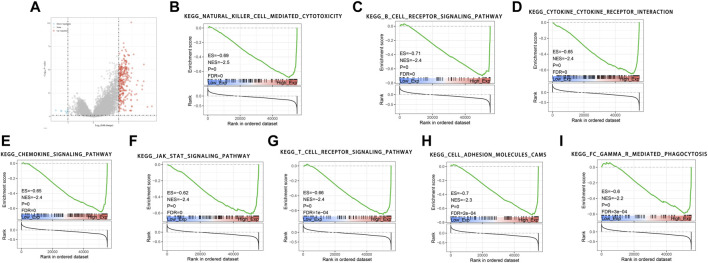
GSEA pathways enriched in samples with high lymphoid-restricted membrane protein (*LRMP*) expression. Two hundred and fifty-seven genes were significantly upregulated and eight genes were significantly downregulated in the high-expression *LRMP* group **(A)**. The results indicate that the natural killer cell-mediated cytotoxicity **(B)**, B cell receptor signaling pathway **(C)**, cytokine-cytokine receptor interaction **(D)**, chemokine signaling pathway **(E)**, JAK-STAT signaling pathway **(F)**, T cell receptor signaling pathway **(G)**, cell adhesion molecules CAMs **(H)**, FC gamma R mediated phagocytosis **(I)** were significantly enriched in lung adenocarcinoma (LUAD) samples with high *LRMP* expression.

We found that 257 genes were significantly upregulated and eight genes were significantly downregulated in the high-expression *LRMP* group compared with the low-expression *LRMP* group (Figure 4A). To investigate the potential molecular function of LRMP in the development of lung adenocarcinoma, we performed GSEAs between samples with low and high *LRMP* expression to predict LRMP-related signaling pathways. Among 186 pathways, 103 signaling pathways were upregulated, and 15 pathways were significantly enriched at NOM *p* < 0.05, FDR <0.01, FWER <0.01, and NES >2 (Table 3). Among the terms associated with immune and inflammatory responses that were enriched and significantly upregulated in the high LRMP group were “natural killer cell-mediated cytotoxicity,” “B cell receptor signaling pathway,” “cytokine-cytokine receptor interaction,” “chemokine signaling pathway,” “JAK-STAT signaling pathway,” “T cell receptor signaling pathway,” and “FC gamma R mediated phagocytosis.” Additionally, the “cell adhesion molecule CAM” pathway was also enriched in the high LRMP group. A summary of the enrichment results is presented in Figures 4B–I.

### Verification of LRMP Functions in A549 Cell Line

**FIGURE 5:**
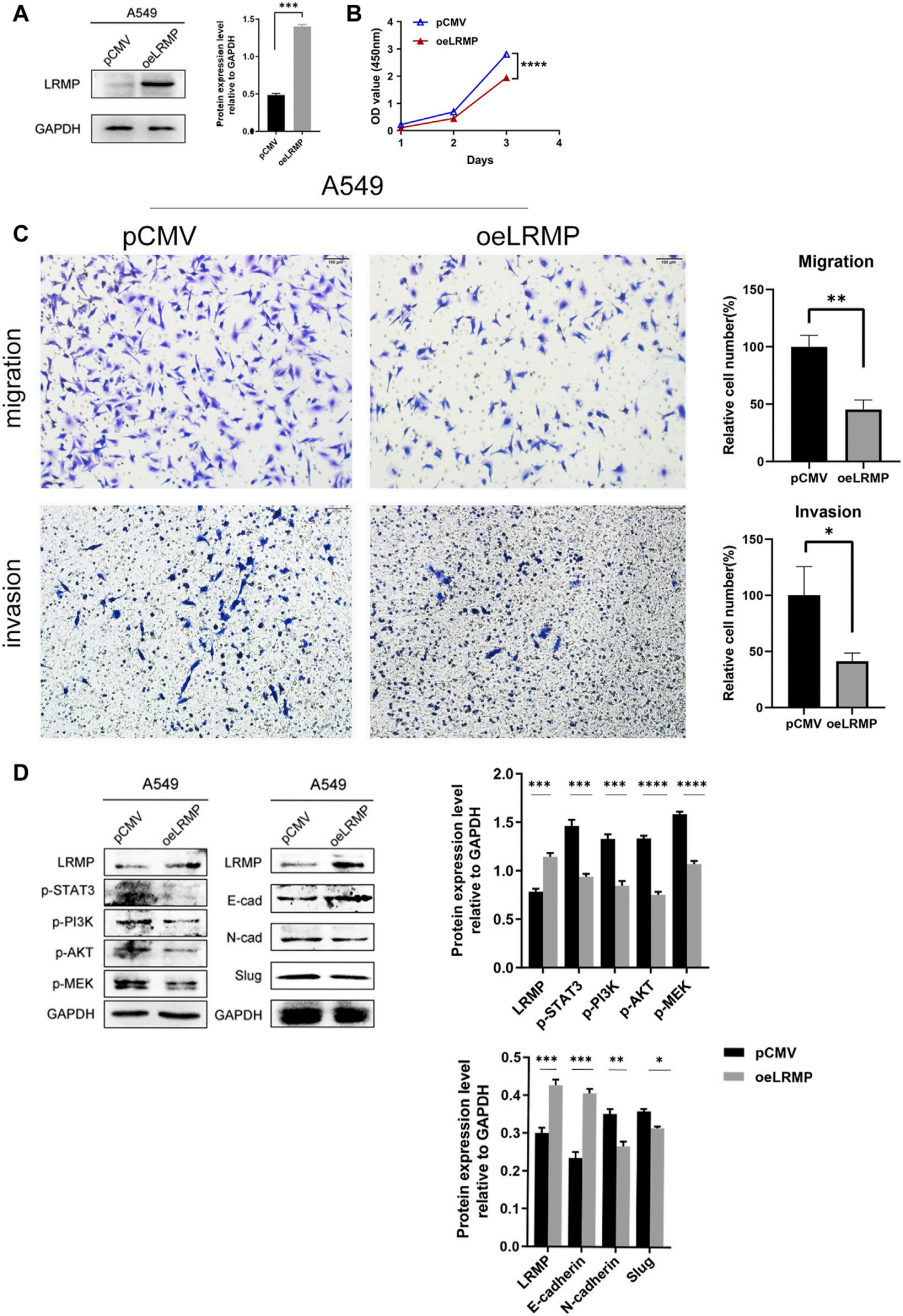
Functional verification and signaling pathway analysis of LRMP in A549 cell line. **(A)** Construction of LRMP overexpression A549. **(B–C)** LRMP inhibited the proliferation, migration and invasion. **(D)** LRMP overexpression downregulated p-PI3K/p-AKT, p-STAT3, p-MEK and EMT signaling pathways by western blotting.

Since LRMP expression was down-regulated in LUAD cell lines, we constructed pCMV- LRMP vector and overexpressed LRMP in A549 cell line to study the function of LRMP in LUAD cell line (Figure5 A). The proliferation capacity of A549 was significantly reduced after overexpression of LRMP by cell counting kit-8 assay (Figure 5B). Furthermore, after overexpression of LRMP, the migration and invasion ability of A549 were also significantly decreased (Figure 5C).

In order to further investigate the mechanism, we selected some pathways for verification according to the bioinformatics analysis. It revealed that p-PI3K/p-AKT, P-STAT3 and P-MEK were down-regulated after overexpression of LRMP by western blotting. N-cadherin and Slug were also down-regulated, and E-cadherin was up-regulated in the EMT pathway (Figure 5D). The alterations of these oncogenic pathways may explain why the proliferation, migration and invasion abilities of A549 are reduced after overexpression of LRMP.

### Analysis of Genes Co-expressed With LRMP in LUAD

**FIGURE 6:**
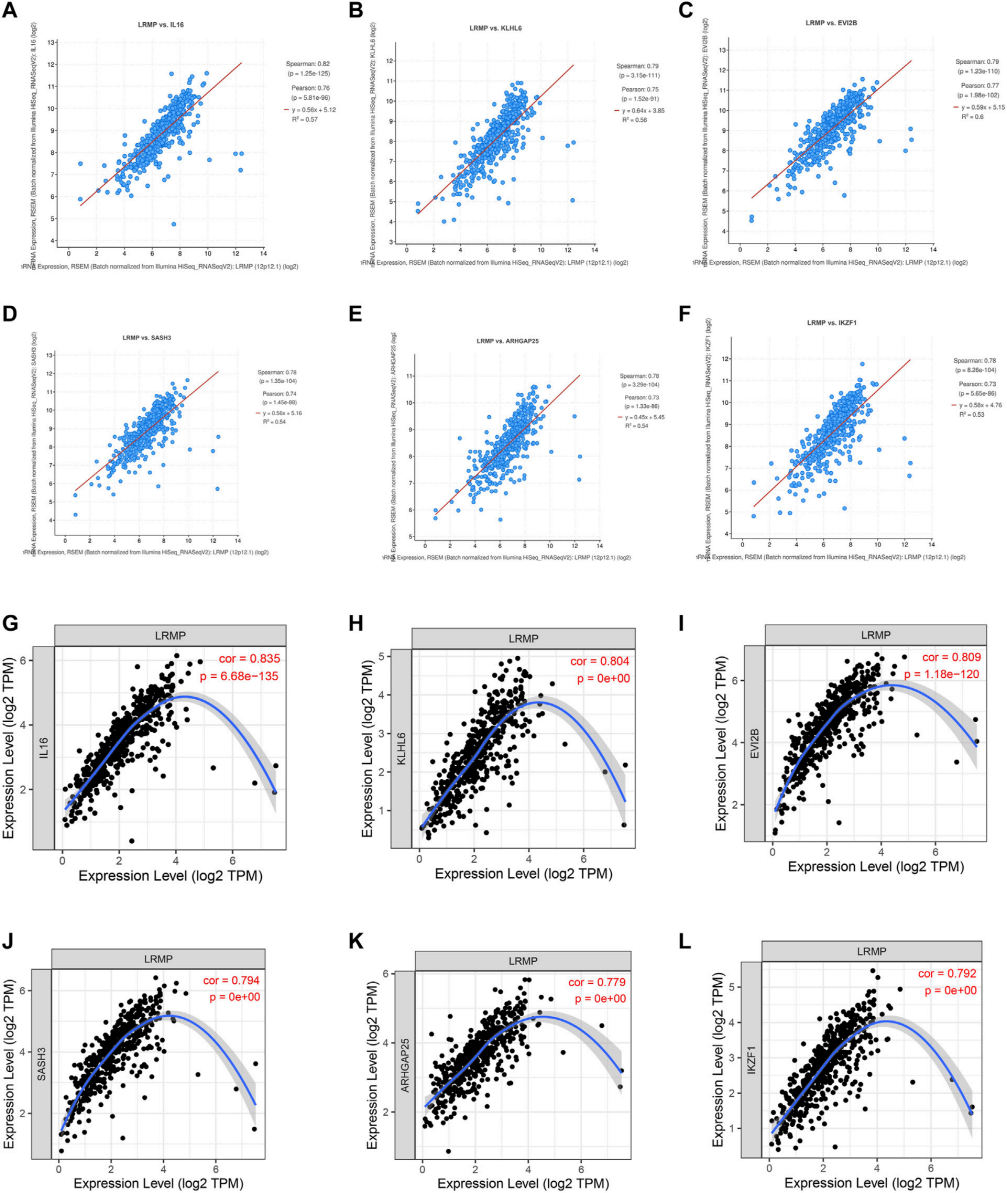
Co-expressed genes of lymphoid-restricted membrane protein (*LRMP*) in lung adenocarcinoma (LUAD). **(A–F)** The genes co-expressed with *LRMP* in LUAD were assessed in cBioPortal database. **(G–L)** *LRMP* was significantly associated with *IL16* (r = 0.835, *p* = 6.68e−135), *KLHL6* (r = 0.804, *p* = 0e + 00), *EVI2B* (r = 0.809, *p* = 1.18e−120), *SASH3* (r = 0.794, *p* = 0e + 00), *ARHGAP25* (r = 0.779, *p* = 0e + 00), and *IKZF1* (r = 0.792, *p* = 0e + 00) in LUAD [*via* Tumor Immune Estimation Resource (TIMER) database].

To further investigate the tumor suppressor effect of LRMP in LUAD, we used cBioPortal to identify the genes positively related to the co-expression of *LRMP* with TCGA data. We selected six genes that were most significantly associated with LRMP (Figures 6A–F). We further verified the correlation between *LRMP* and these genes using TIMER. The results showed that LRMP was significantly correlated with IL16 (r = 0.835, *p* = 6.68e−135), KLHL6 (r = 0.804, *p* = 0e+00), EVI2B (r = 0.809, *p* = 1.18e−120), SASH3 (r = 0.794, *p* = 0e + 00), ARHGAP25 (r = 0.779, *p* = 0e + 00), and IKZF1 (r = 0.792, *p* = 0e + 00) (Figures 6G–L). Additionally, we found that the genes co-expressed with *LRMP* were downregulated in LUAD (Supplementary Figure S2A). The genes co-expressed with *LRMP* were associated with the LUAD stages (Supplementary Figure S2B). Furthermore, high expression of *LRMP*-related genes was significantly associated with better OS in patients with LUAD, except for KLHL6 (Supplementary Figure S2C). This suggests that *LRMP* and its co-expressed genes contribute to the inhibition of lung adenocarcinoma carcinogenesis, resulting in better survival in patients with LUAD.

In order to investigate whether LRMP directly regulated these six co-expressed genes, we used qRT-PCR to detect the mRNA levels of these six genes after overexpressing LRMP in A549. We found there was no significant changes in other five genes except KLHL6 (Supplementary Figures S3A,B). The results suggest that these co-expressed genes may influence the expression level of LRMP, or LRMP and these co-expressed genes may be jointly regulated by other mechanisms. The relationship between LRMP and co-expressed genes needs further studies.

### LRMP and Its Co-Expressed Genes Are Significantly Associated With Tumor-Infiltrating Immune Cells in LUAD

**FIGURE 7:**
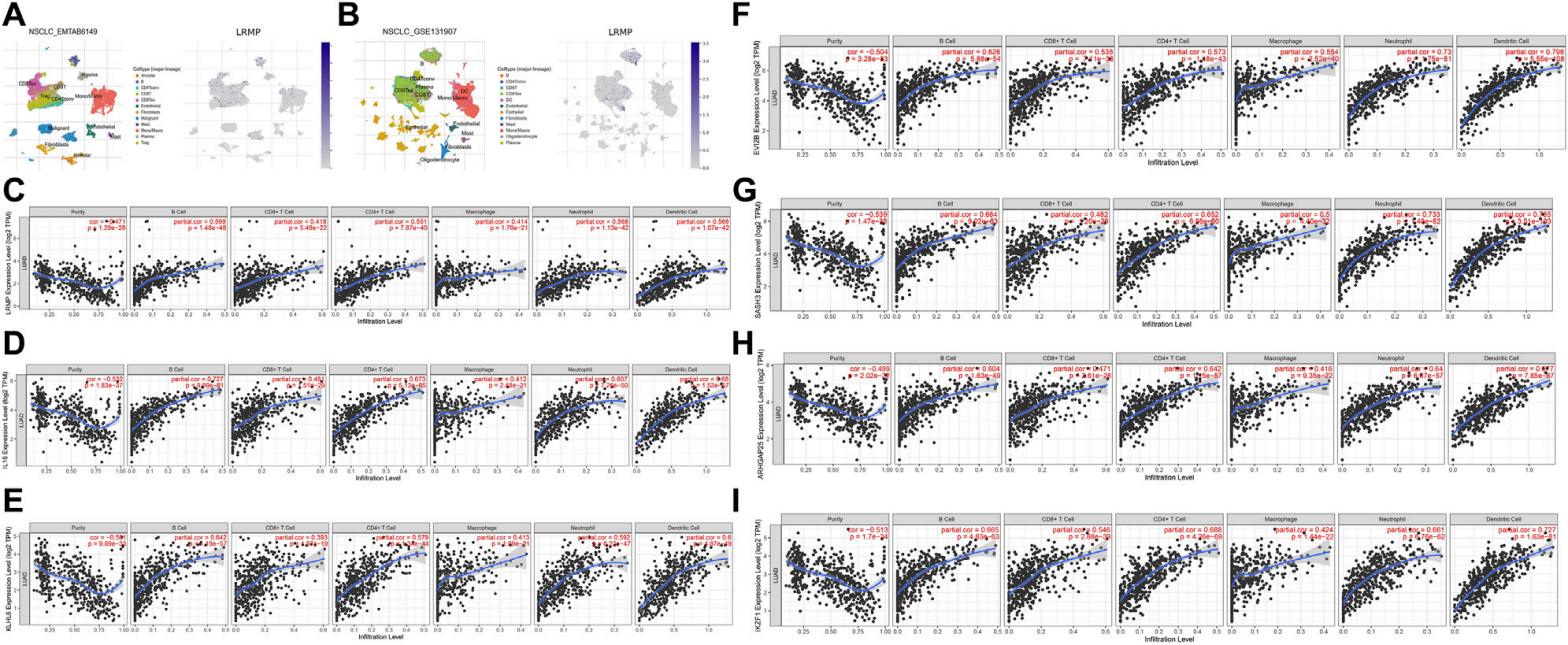
Correlation of lymphoid-restricted membrane protein (*LRMP*) and its co-expressed genes with tumor-infiltrating immune cell in lung adenocarcinoma (LUAD). **(A–B)** Single-cell distribution of LRMP in the LUAD microenvironment in EMTAB6149 and GSE 131907 datasets. **(C–I)** Correlation of *LRMP, IL16, KLHL6, EVI2B, SASH3, ARHGAP25*, and *IKZF1* expression with tumor-infiltrating- immune in LUAD.

We used TISCH to investigate the expression of *LRMP* in the LUAD tumor microenvironment at the single-cell level using datasets EMTAB6149 and GSE131907 (Figures 7A,B). In EMTAB6149, *LRMP* was mainly expressed in immune cells, including B cells, plasma cells, CD8T ex cells, CD8T cells, CD4Tconv cells, mono/macrophages, mast cells, and regulatory T cells. *LRMP* was slightly expressed in malignant cells; however, it was almost not expressed in endothelial cells, fibroblasts, and alveolar cells. In GSE131907, LRMP was mainly expressed in B cells, CD4Tconv cells, plasma cells, CD8T cells, CD8Tex cells, dendritic cells, mono/macrophages, and mast cells, whereas it was almost not expressed in endothelial cells, fibroblasts, epithelial cells, and oligodendrocytes. Furthermore, we used the TIMER database to explore the relationship between *LRMP* expression and immune cell infiltration. TIMER showed that *LRMP* expression was positively correlated with several types of infiltrating immune cells, including B cells (r = 0.599, *p* = 1.48e−48), CD8^+^ T cells (r = 0.418, *p* = 5.48e−22), CD4^+^ T cells (r = 0.551, *p* = 7.87e−40), macrophages (r = 0.414, *p* = 1.76e−21), neutrophils (r = 0.568, *p* = 1.13e−42), and dendritic cells (r = 0.566, *p* = 1.07e−42) (Figure 7C).

Moreover, the relationship between genes co-expressed with *LRMP* and infiltrating immune cells was also analyzed. We found that *LRMP*-related *genes IL16, KLHL6, EVI2B, SASH3, ARHGAP25*, and *IKZF1* were also significantly associated with six types of immune cells. *IL16* expression was positively associated with B cells (r = 0.727, *p* = 9.99e−81), dendritic cells (r = 0.68, *p* = 1.53e−67), CD4^+^ T cells (r = 0.673, *p* = 5.13e−65), neutrophils (r = 0.607, *p* = 7.26e−50), CD8^+^ T cells (r = 0.481, *p* = 1.51e−29), and macrophages (r = 0.412, *p* = 2.48e−21) (Figure 7D). *KLHL6* expression was correlated with B cells (r = 0.642, *p* = 1.19e−57), dendritic cells (r = 0.6, *p* = 4.87e−49), neutrophils (r = 0.592, *p* = 6.22e−47), CD4^+^ T cells (r = 0.579, *p* = 1.34e−44), macrophages (r = 0.413, *p* = 1.89e−21), and CD8^+^ T cells (r = 0.393, *p* = 1.87e−19) (Figure 7E). *EVI2B* expression was significantly associated with dendritic cells (r = 0.796, *p* = 5.55e−108), neutrophils (r = 0.73, *p* = 1.75e−81), B cells (r = 0.626, *p* = 5.88e−54), CD4^+^ T cells (r = 0.573, *p* = 1.18e−43), macrophages (r = 0.554, *p* = 2.52e−40), and CD8^+^ T cells (r = 0.538, *p* = 7.11e−38) (Figure 7F). *SASH3* expression was positively correlated with dendritic cells (r = 0.785, *p* = 3.51e−103), neutrophils (r = 0.733, *p* = 1.40e−82), B cells (r = 0.664, *p* = 9.02e−63), CD4^+^ T cells (r = 0.652, *p* = 6.98e−60), macrophages (r = 0.5, *p* = 4.40e−32), and CD8^+^ T cells (r = 0.482, *p* = 1.20e−29) (Figure 7G). *ARHGAP25* expression was associated with dendritic cells (r = 0.677, *p* = 7.85e−67), CD4^+^ T cells (r = 0.642, *p* = 1.15e−57), neutrophils (r = 0.64, *p* = 6.67e−57), B cells (r = 0.604, *p* = 1.63e−49), CD8^+^ T cells (r = 0.471, *p* = 2.61e−28), and macrophages (r = 0.416, *p* = 9.35e−22) (Figure 7H). Furthermore, *IKZF1* expression was significantly correlated with the levels of infiltrating immune cells, including dendritic cells (r = 0.727, *p* = 1.63e−81), CD4^+^ T cells (r = 0.688, *p* = 4.26e−69), B cells (r = 0.665, *p* = 4.83e−63), neutrophils (r = 0.661, *p* = 6.76e−62), CD8^+^ T cells (r = 0.546, *p* = 2.88e−39), and macrophages (r = 0.424, *p* = 1.44e−22) (Figure 7I). This suggests that *LRMP* and its co-expressed genes may participate in the immune response in the tumor microenvironment by affecting immune cells.

**FIGURE 8:**
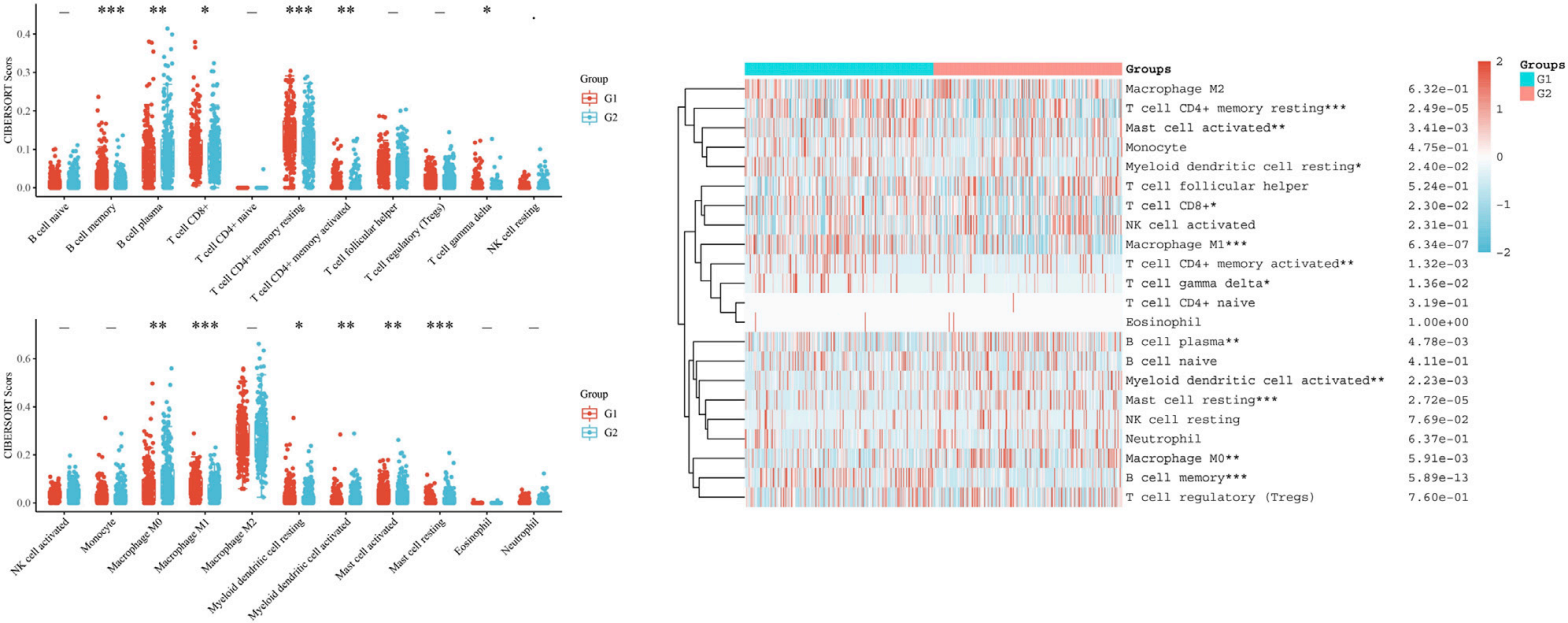
CIBERSORT analysis of TCGA-lung adenocarcinoma (LUAD) dataset. Twenty-two kinds of tumor-infiltrating immune cells are plotted according to lymphoid-restricted membrane protein (*LRMP*) expression level. There were significant differences in memory B cell, plasma B cell, CD8^+^ T cell, resting memory CD4^+^ T cell, activated memory CD4^+^ T cell, gamma delta T cell, M0 macrophage, M1 macrophage, resting myeloid dendritic cell, activated myeloid dendritic, activated mast cell, and resting mast cell (*p < 0.05, **p < 0.01, ***p < 0.001).

Additionally, CIBERSORT analysis divided LUAD patients into two groups: the G1 group with high *LRMP* expression and the G2 group with low LRMP expression. The results showed that there were differences in the number of infiltrating immune cells between the two groups, including resting memory CD4^+^ T cells (*p* = 2.49e−05), activated mast cells (*p* = 3.41e−03), resting myeloid dendritic cells (*p* = 0.024), CD8^+^ T cells (*p* = 0.023), M1 macrophages (*p* = 6.34e−07), activated memory CD4^+^ T cells (*p* = 1.32e−03), gamma delta T cells (*p* = 0.0136), plasma B cells (*p* = 4.78e−03), activated myeloid dendritic cells (*p* = 2.23e−03), resting mast cells (*p* = 2.72e−05), M0 macrophages (*p* = 5.91e−03), and memory B cells (*p* = 5.89e−13) (Figure 8) The correlation of *LRMP* and gene markers of several immune cells were listed in Supplementary Table S1. Overall, *LRMP* and its co-expressed genes were associated with tumor-infiltrating immune cells in LUAD.

### LRMP Expression Was Correlated With Immune Checkpoints in LUAD

**FIGURE 9:**
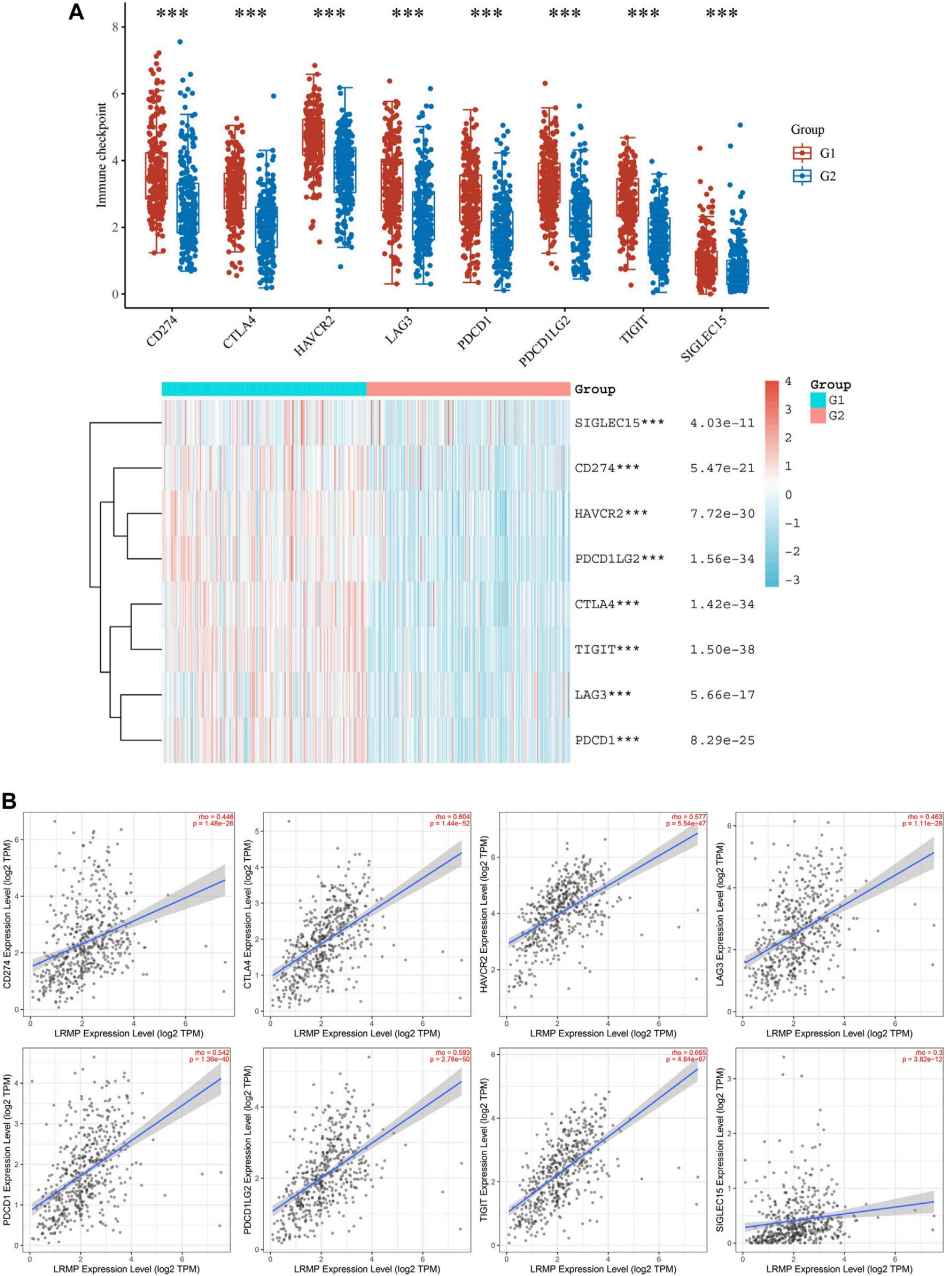
Correlation of lymphoid-restricted membrane protein (*LRMP*) expression with immune checkpoints in lung adenocarcinoma (LUAD). **(A)** The difference in immune checkpoint expression between the two groups divided by *LRMP* expression (*p < 0.05, **p < 0.01, ***p < 0.001). (B) The relationship between LRMP and immune checkpoints is demonstrated using a scatter plot in Tumor Immune Estimation Resource (TIMER) 2.0.

We found a significant positive correlation between *LRMP* and several immune cells (Figure 7). The CIBERSORT results showed that CD8^+^ T cells and M1 type macrophages were significantly upregulated in the group with high *LRMP* expression (Figure 8), indicating that LRMP may be related to immune checkpoints. To further investigate the relationship between LRMP and immune checkpoints, we divided patients with LUAD into two groups and compared the expression levels of immune checkpoints in the two groups. The G1 group had a high expression of *LRMP*, whereas the G2 group had a low expression of *LRMP*. As shown in Figure 9A, the immune checkpoints in the G1 group were highly expressed, includingCD274 (PD-L1) (*p* = 5.47e−21), CTLA4 (*p* = 1.42e−34), HAVCR2 (*p* = 7.72e−30), LAG3 (*p* = 5.66e−17), PDCD1 (PD-1) (*p* = 8.29e−25), PDCD1LG2 (*p* = 1.56e−34), TIGIT (*p* = 1.50e−38), and SIGLEC15 (*p* = 4.03e−11).

Furthermore, we also verified the relationship between LRMP and immune checkpoints using TIMER 2.0 (Figure 9B). As the results showed, *LRMP* expression was related to CD274 (r = 0.446, *p* = 1.48e−26), CTLA4 (r = 0.604, *p* = 1.44e−52), HAVCR2 (r = 0.577, *p* = 5.54e−47), LAG3 (r = 0.463, *p* = 1.11e−28), PDCD1 (r = 0.542, *p* = 1.36e−40), PDCD1LG2 (r = 0.593, *p* = 2.78e−50), TIGIT (r = 0.665, *p* = 4.84e−67), and SIGLEC15 (rho = 0.3, *p* = 3.82e−12). Altogether, these results suggest that the expression of LRMP is related to immune checkpoints and may be suggestive of immunotherapy. In order to verify the relationship between LRMP and PDL1(CD274), we used qRT-PCR to detect the mRNA expression level of PDL1(CD274) in A549 cell line after the overexpression of LRMP, and found that the expression level of PDL1 was increased after overexpression of LRMP, but there was no statistical difference (Supplementary Figure S4).

## Discussion

Abnormal gene expression is associated with tumorigenesis and indicates the prognosis of patients with lung cancer (Ali et al., 2021; Shen et al., 2021). However, the molecular mechanisms underlying LUAD carcinogenesis and tumor microenvironment interaction remain unclear. In this study, we demonstrated that *LRMP* expression was significantly decreased in LUAD tissue and was associated with vital status, age, sex, TNM stage, and new tumor event type in patients with LUAD. Furthermore, low *LRMP* expression is associated with poor prognosis and overall survival. These results suggest that LRMP may be a tumor suppressor gene that is involved in the progression of lung adenocarcinoma.

Previous studies have reported that LRMP plays an important role in regulation and antigen receptor transportation (Behrens et al., 1994; Snyder et al., 1997). However, the role of LRMP in tumorigenesis remains unclear. In animal studies, *LRMP* polymorphism increased the risk of lung tumors; however, this effect was not significant in humans (Manenti et al., 2004; Manenti and Dragani, 2005; Manenti et al., 2006). Zhang et al. reported that LRMP is a favorable factor in DLBCL (Zhang et al., 2007). In this study, we found that *LRMP* was abnormally expressed in a variety of cancers, indicating that it may play a role in tumorigenesis. In LUAD, we demonstrated that *LRMP* expression was significantly lower in tumor tissues than in normal tissues, which suggests that LRMP may be involved in the inhibition of tumorigenesis in LUAD. In addition, we also verified the decreased expression of LRMP in LUAD cell lines and tumor tissues.

To further explore the molecular function and possible mechanisms of LRMP in LUAD tumorigenesis, we performed GSEA analysis to explore the pathways enriched in samples with high *LRMP* expression. Fifteen pathways related to cell adhesion and immune and inflammatory responses were selected (Supplementary Table S2). Some cell adhesion molecules, which have been identified as tumor suppressor genes, are associated with a better prognosis in cancers (Sawada et al., 2020; Chao et al., 2021). Immune-and inflammation-related pathways such as “natural killer cell-mediated cytotoxicity,” “B cell receptor signaling pathway,” “cytokine-cytokine receptor interaction,” “chemokine signaling pathway,” “JAK-STAT signaling pathway,” “T cell receptor signaling pathway” and “FC gamma R mediated phagocytosis,” were also enriched in the *LRMP* high expression group. Natural killer cells and cytotoxic T cells play key roles in tumor immunity for tumor elimination. Natural killer cell-mediated cytotoxicity is an important anti-tumor mechanism (Herberman et al., 1975; Ojo and Wigzell, 1978). T cells recognize antigenic peptides presented by MHC molecules through the T cell receptor signaling pathway, which plays an anti-tumor role (Germain, 1994). The JAK-STAT pathway is involved in the formation of certain tumors and regulates T cell survival and function (Villarino et al., 2017; Zhu et al., 2019). Additionally, cytokine-and chemokine-related pathways may influence the composition of the tumor microenvironment. Altogether, these results suggest that LRMP may be involved in anti-tumor immune responses that play a role in inhibiting tumorigenesis, leading to a better prognosis in patients with LUAD. Through *in vitro* experimental studies, we confirmed that the overexpression of LRMP could decrease the proliferation, migration and invasion in A549 cells. We also found that some oncogenic signaling pathways, including p-STAT3, p-PI3K-p-AKT, p-MEK and EMT pathways, were all downregulated after LRMP overexpression. These experimental data further verified the results of web-based bioinformatics analysis and indicated that LRMP may act as a tumor suppressor gene in LUAD.

Co-expression analysis of TIMER and cBioPortal showed that *LRMP* was strongly correlated with *IL16, KLHL6, EVI2B, SASH3, ARHGAP25*, and *IKZF1*, which are involved in tumorigenesis or immune/inflammatory response (Yang et al., 2017; Choi et al., 2019; Xu et al., 2019; Wang et al., 2020; Mougiakakos et al., 2021; Yang et al., 2021). Our results also showed that *LRMP*-related gene expression was reduced in LUAD, which was associated with a worse prognosis in patients with LUAD. Additionally, our results suggest that *LRMP* co-expression genes are significantly associated with tumor-infiltrating immune cells. The immune response is assumed to be critical for the development and progression of LUAD. This implies that an improved tumor immune response may benefit LUAD patients, resulting in improved clinical symptoms and OS (Somasundaram and Burns, 2017).

The composition of tumor-infiltrating immune cells was assessed using CIBERSORT analysis based on *LRMP* expression. We found that the expression of 12 types of immune cells, including memory B cells, plasma B cells, CD8^+^ T cells, resting memory CD4^+^ T cells, activated memory CD4^+^ T cells, gamma delta T cells, M0 macrophages, M1 macrophages, resting myeloid dendritic cells, activated myeloid dendritic cells, activated mast cells, and resting mast cells, were significantly different between different *LRMP* expression. These data suggest that LRMP is closely associated with tumor-infiltrating immune cells. Moreover, *LRMP* and its co-expressed genes may contribute to the immune response in LUAD, leading to a better prognosis. The results of CIBERSORT showed that the expression of M1 macrophages in the low *LRMP* expression group was lower than that in the high *LRMP* expression group. However, there was no significant difference in M2 macrophages between the different *LRMP* expression groups. There was also a positive correlation between *LRMP* and the marker genes of M1 macrophages. Previous studies have reported that polarization of M1 macrophages could inhibit tumor proliferation, invasion, metastasis, and angiogenesis, as well as promote apoptosis. M1 macrophage densities in tumor islets and stroma were positively correlated with the survival time of patients with NSCLC (Ma et al., 2010; Ye et al., 2018). Our findings suggest that high *LRMP* expression may upregulate the polarization of macrophages to M1 macrophages, which could contribute to the inhibition of LUAD tumorigenesis. Additionally, there were more CD8+T cells in the group with high *LRMP* expression and more M0 macrophages in the group with low *LRMP* expression. CD8 + T cells are involved in the killing of tumor cells (Sasidharan Nair and Elkord, 2018). Increased M0 macrophage composition is associated with a poorer prognosis in patients with cancer (Farha et al., 2020; Jairath et al., 2020). These data indicated that LUAD with high expression of LRMP may have a higher level of anti-tumor immune cell infiltration and a more positive tumor immune microenvironment. These results explain the relationship between LRMP and LUAD prognosis from the perspective of immune infiltration. Furthermore, due to the differences in immune cell composition between the two groups with high and low *LRMP* expression, we further explored the differences in immune checkpoints between the two groups. We found that the expression level of immune checkpoints was also higher in the group with high *LRMP* expression, and there was a significant positive correlation between LRMP and immune checkpoints, including CD274 (PD-L1), CTLA4, HAVCR2, LAG3, PDCD1 (PD-1), PDCD1LG2, TIGIT, and SIGLEC15 (Doyle et al., 2001; Keir et al., 2007; Weber, 2010; Li et al., 2015; Janakiram et al., 2016; Zhu et al., 2016; Sun et al., 2021b). PDL1 (CD274) has been used as a biomarker to predict the response to immunotherapy. In our study, it was found that LRMP was positively correlated with PDL1 (CD274), and PDL1 (CD274) was increased after overexpression of LRMP. Therefore, these results suggest that patients in the group with high *LRMP* expression may have a better response to immunotherapy than those with low *LRMP* expression.

Although our findings demonstrated that LRMP was associated with immune infiltrates and acted as a prognostic biomarker in lung adenocarcinoma, this study has some limitations. First, our study was based on limited data samples, which may have some bias. Second, whether the findings could apply to all LUAD patients with different molecular alterations still needs to be confirmed.

In conclusion, *LRMP* expression was significantly reduced in patients with LUAD and indicated a poor prognosis. Overexpression of LRMP could decrease the proliferation, migration and invasion, as well as inhibit multiple oncogenic signaling pathways in LUAD. LRMP expression was significantly associated with the levels of immune cell infiltration and immune checkpoint expression. Therefore, LRMP may act as a tumor suppressor gene and an indicator of the immunotherapy response. Further studies are needed to validate these findings.

## Data Availability

Publicly available datasets were analyzed in this study. This data can be found here: The Cancer Genome Atlas (TCGA) (https://www.cancer.gov/tcga) GEPIA2 (http://gepia2.cancer-pku.cn/) The Human Protein Atlas (HPA) (https://www.proteinatlas.org/) The Kaplan Meier plotter (http://kmplot.com/analysis/) Tumor Immune Single-cell Hub (TISCH) (http://tisch.comp-genomics.org) Tumor Immune Estimation Resource (TIMER) (https://cistrome.shinyapps.io/timer/) TIMER 2.0 (http://timer.cistrome.org/) cBioPortal database (https://www.cbioportal.org/) and TISIDB database (http://cis.hku.hk/TISIDB/).

## Supplementary Material

Supplementary Figure S1
**(A)** LRMP mRNA level in different stages of LUAD. **(B-C)** LRMP mRNA level analysis according to EGFR or ALK mutation and wild type.

Supplementary Figure S2
**(A)** Lymphoid-restricted membrane protein (LRMP) co-expression genes expression in different cancer types. **(B)** Relationship between LRMP co-expressed genes and TNM stage in lung adenocarcinoma (LUAD). **(C)** Analysis of LRMP co-expressed genes in LUAD overall survival.

Supplementary Figure S3
**(A)** LRMP mRNA level was upregulated after transfection in A549 cells. **(B)** Changes of genes co-expressed with LRMP in overexpressed A549 cell lines.

Supplementary Figure S4 PD-L1 (CD274) expression inA549 cells with LRMP overexpression.

Supplementary Table S1 Correlation of LRMP and gene markers in immune cells.

Supplementary Table S2 GSEA pathways upregulationin high expression of LRMP.
