# Supplementary figures and images for: LRMP Associates With Immune Infiltrates and Acts as a Prognostic Biomarker in Lung Adenocarcinoma
Source: Front Mol Biosci. 2021 Nov 26;8:711928. doi: 10.3389/fmolb.2021.711928 (PMC8661541; doi:10.3389/fmolb.2021.711928)

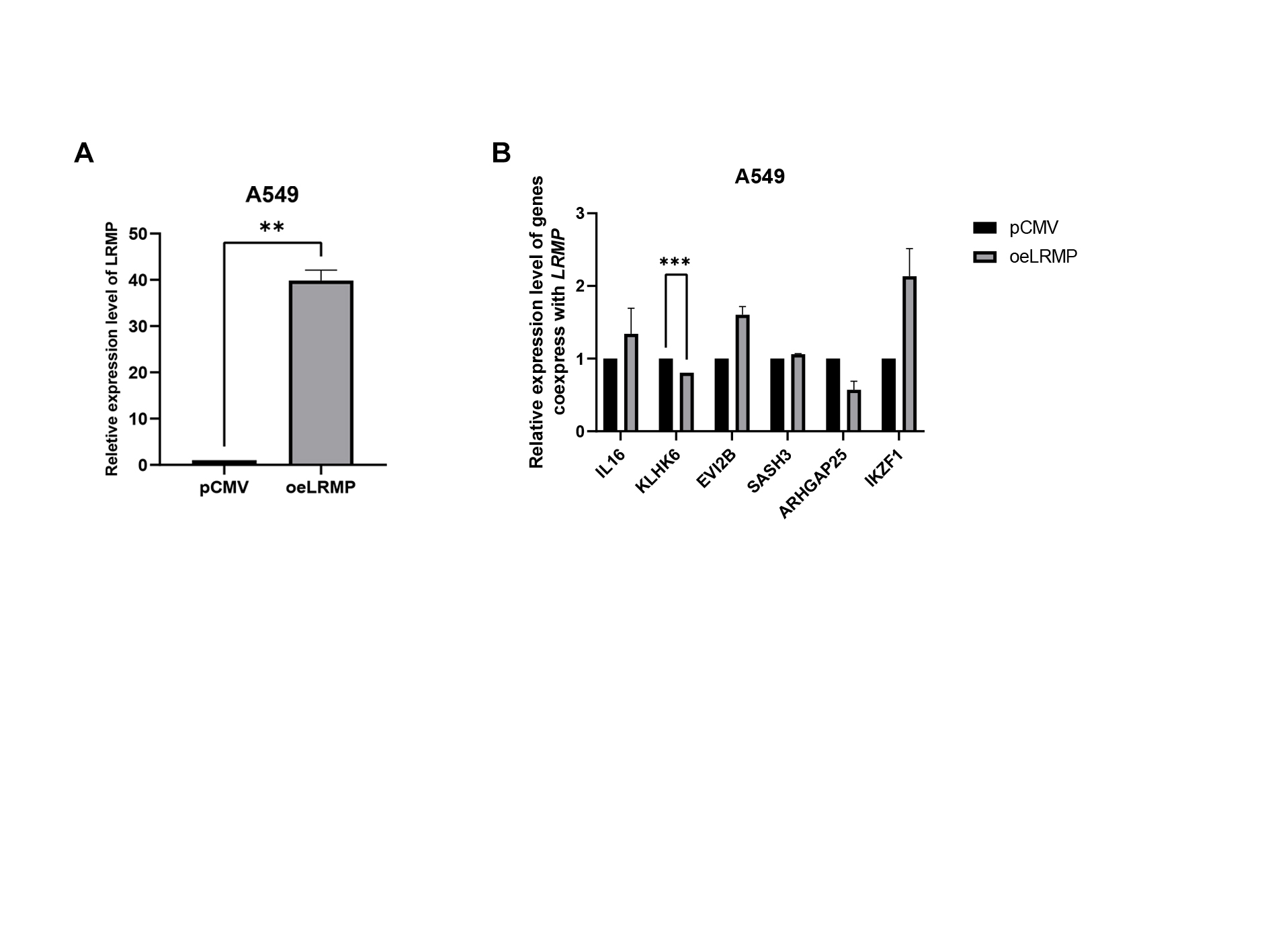

Supplement: Supplementary file 1 [file Image3.JPEG]

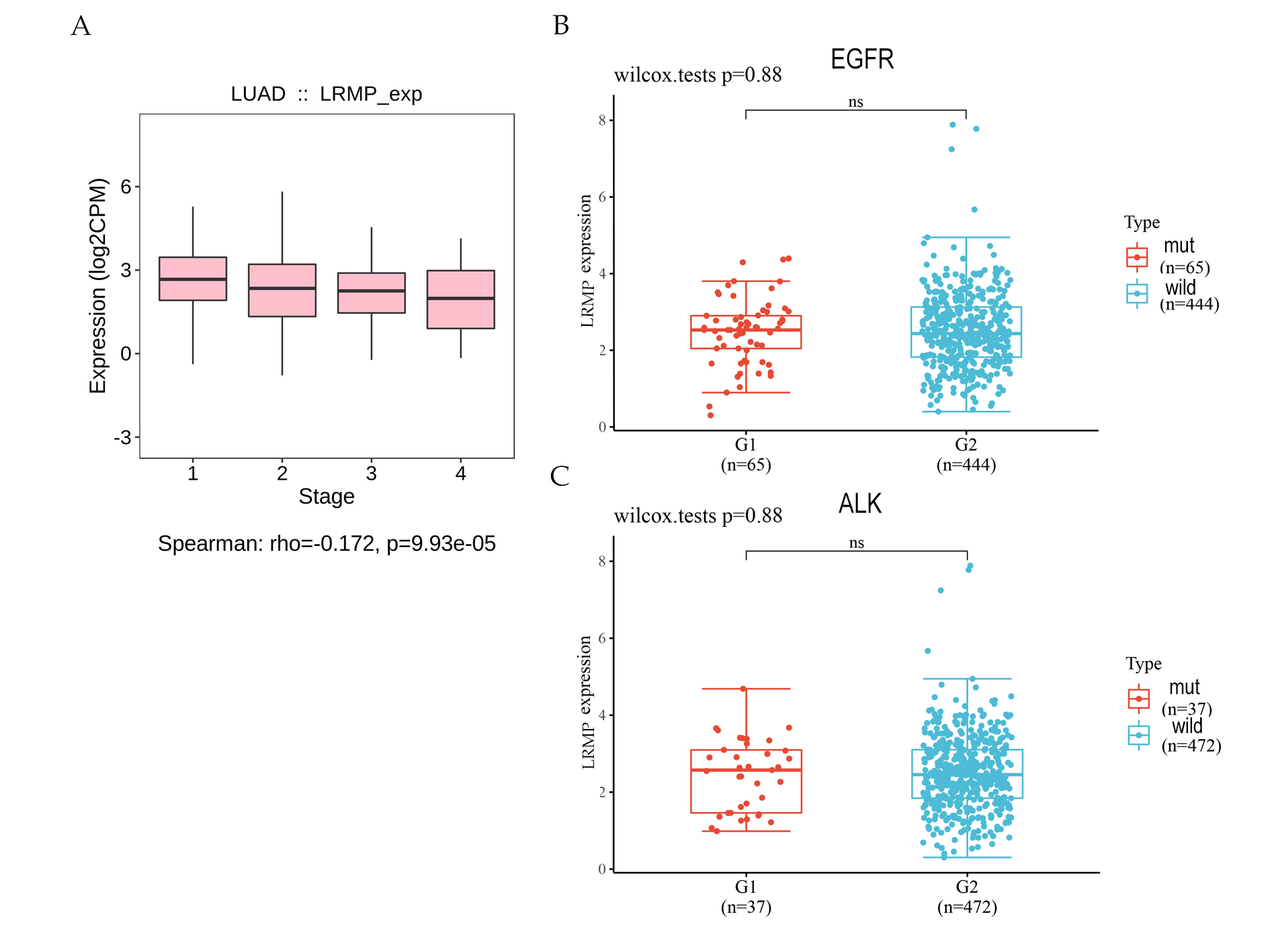

Supplement: Supplementary file 2 [file Image1.JPEG]

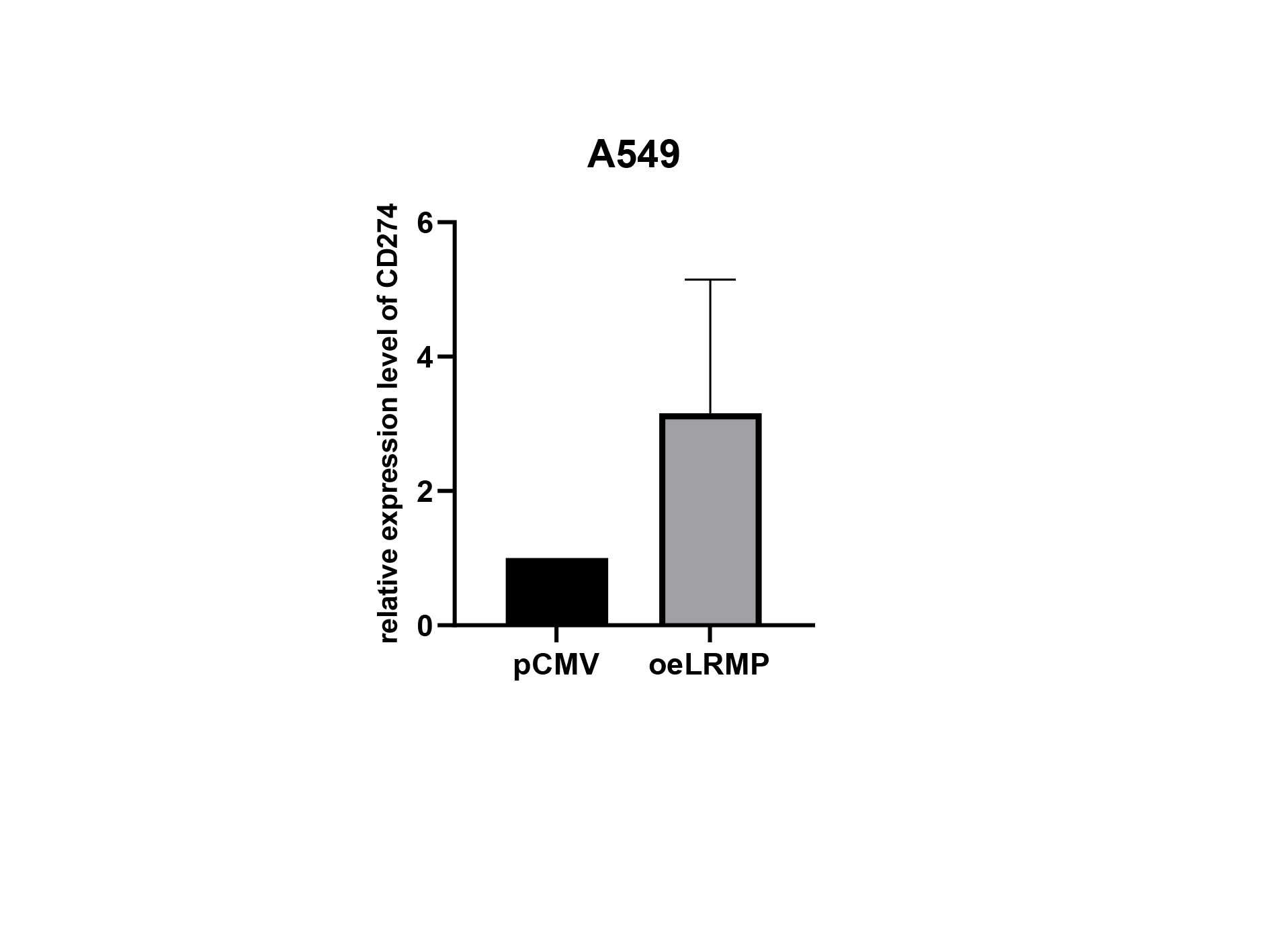

Supplement: Supplementary file 3 [file Image4.JPEG]

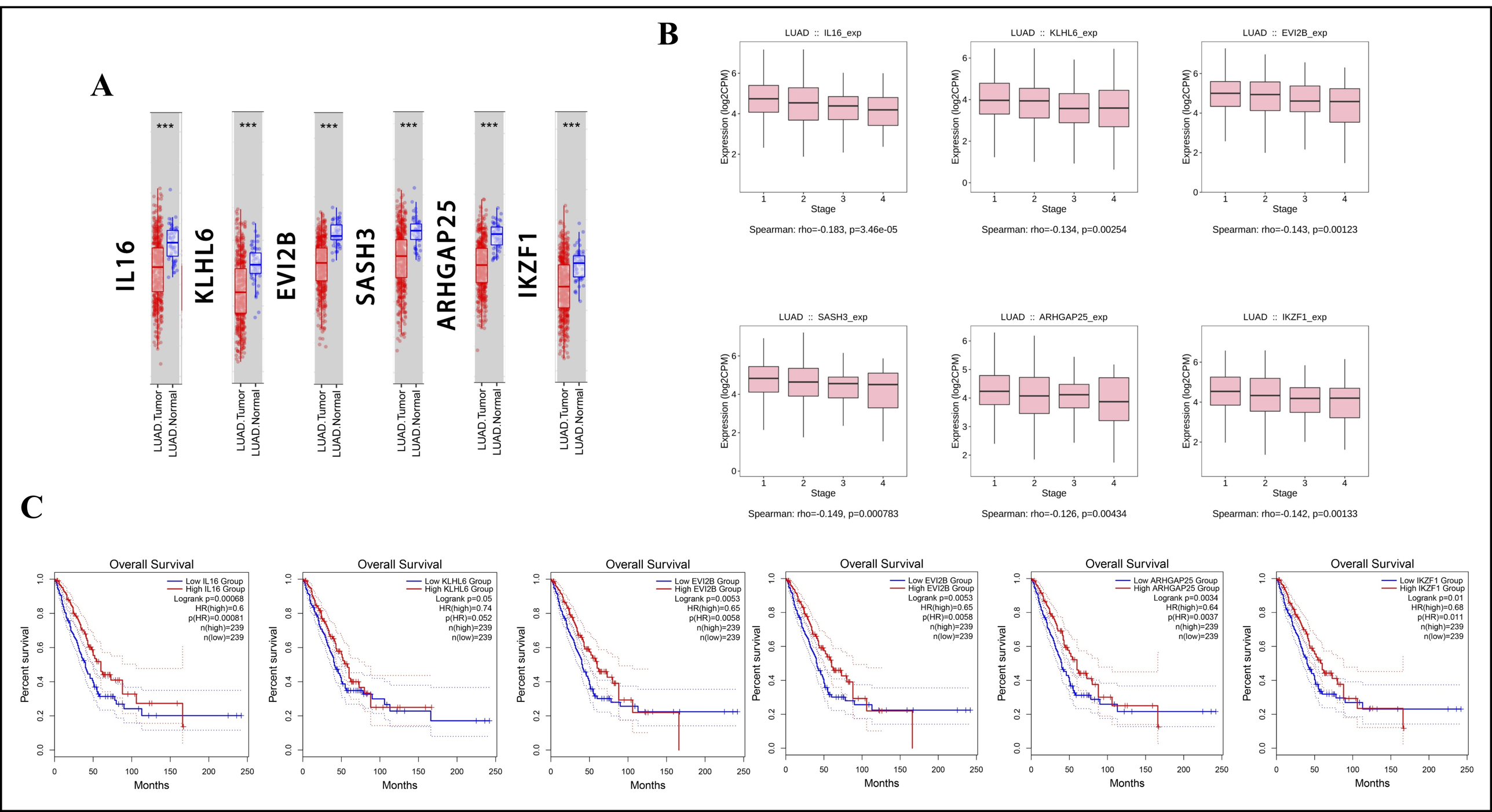

Supplement: Supplementary file 4 [file Image2.JPEG]

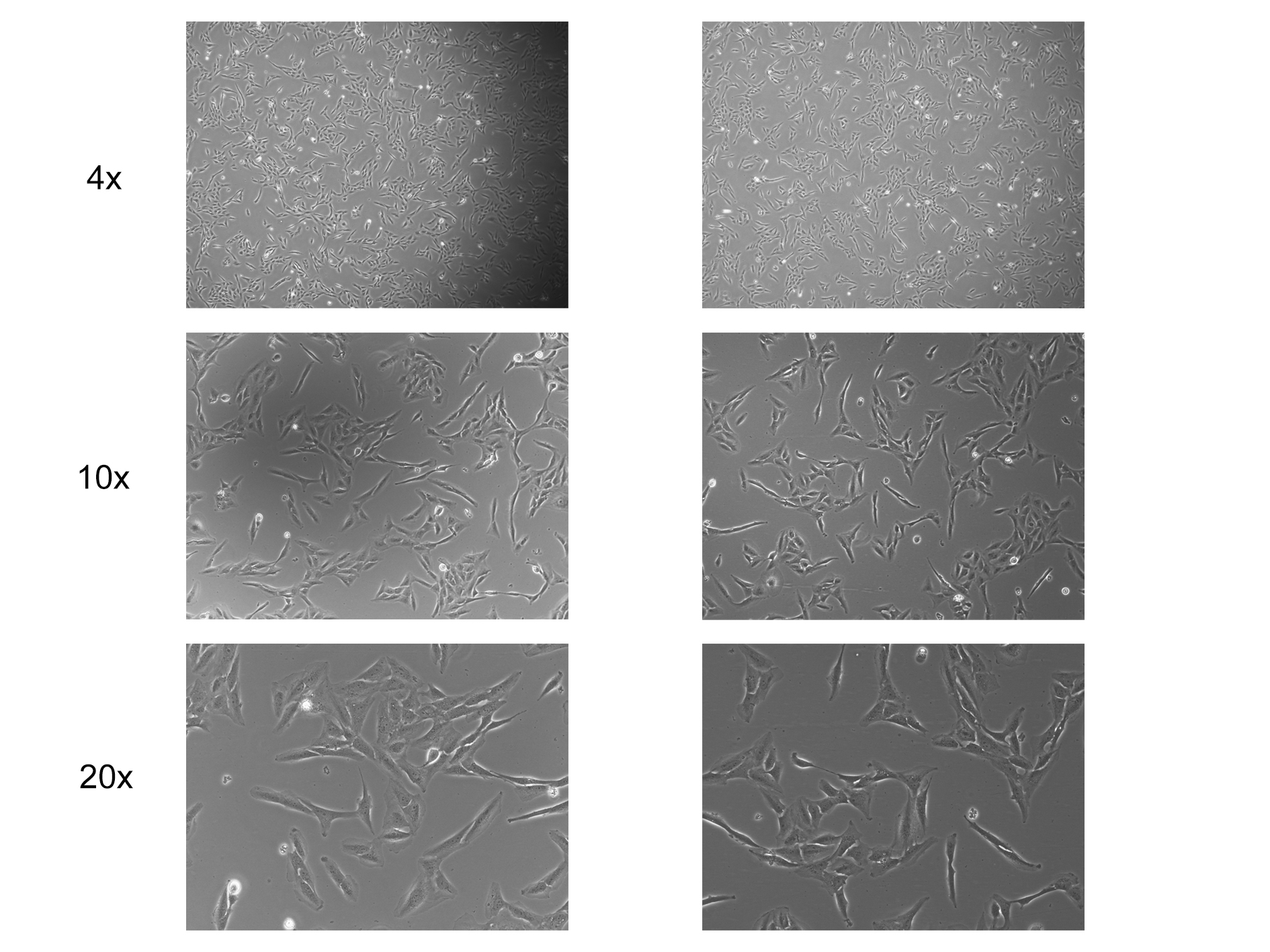

Supplement: Supplementary file 5 [file Image5.JPEG]
