# Supplementary material for: LRMP Associates With Immune Infiltrates and Acts as a Prognostic Biomarker in Lung Adenocarcinoma
Source: Front Mol Biosci. 2021 Nov 26;8:711928. doi: 10.3389/fmolb.2021.711928 (PMC8661541; doi:10.3389/fmolb.2021.711928)
Supplement: Supplementary file 6 [file DataSheet2.docx]

**Supplementary table 1. Correlation of LRMP and gene markers in immune cells**

| **LUAD** | | | | | |
| --- | --- | --- | --- | --- | --- |
| **Description** | **Gene markers** | **None** | | **Purity** | |
|  |  | **cor** | **p-value** | **cor** | **p-value** |
| CD8+T | CD8A | 0.537 | *** | 0.431 | *** |
|  | CD8B | 0.485 | *** | 0.407 | *** |
| T cell (general) | CD3D | 0.626 | *** | 0.516 | *** |
|  | CD3E | 0.693 | *** | 0.602 | *** |
|  | CD2 | 0.689 | *** | 0.6 | *** |
| B cell | CD19 | 0.674 | *** | 0.584 | *** |
|  | CD79A | 0.619 | *** | 0.525 | *** |
| M1 Macrophage | INOS(NOS2) | 0.147 | ** | 0.058 | 0.202 |
|  | IRF5 | 0.404 | *** | 0.319 | *** |
|  | COX2(PTGS2) | -0.036 | 0.412 | -0.052 | 0.25 |
| Dendritic cell | HLA-DPB1 | 0.598 | *** | 0.521 | *** |
|  | HLA-DQB1 | 0.414 | *** | 0.306 | *** |
|  | HLA-DRA | 0.571 | *** | 0.485 | *** |
|  | HLA-DPA1 | 0.573 | *** | 0.498 | *** |
|  | BDCA-1(CD1C) | 0.455 | *** | 0.38 | *** |
|  | BDCA-4(NRP1) | 0.135 | * | 0.103 | 0.0221 |
|  | CD11c(ITGAX) | 0.596 | *** | 0.511 | *** |
| Th1 | T-bet (TBX21) | 0.577 | *** | 0.478 | *** |
|  | STAT4 | 0.527 | *** | 0.408 | *** |
|  | STAT1 | 0.366 | *** | 0.272 | *** |
|  | IFN-γ (IFNG) | 0.366 | *** | 0.265 | *** |
|  | TNF-α (TNF) | 0.444 | *** | 0.32 | *** |
| Th2 | GATA3 | 0.527 | *** | 0.418 | *** |
|  | STAT6 | 0.19 | *** | 0.233 | *** |
|  | STAT5A | 0.655 | *** | 0.587 | *** |
|  | IL13 | 0.209 | *** | 0.133 | * |

*P < 0.01; **P < 0.001; ***P < 0.0001.

**Supplementary table 2. GSEA pathways upregulated due to high expression of LRMP**

| **Term** | **ES** | **NES** | **NP** | **FDR** | **FWER** |
| --- | --- | --- | --- | --- | --- |
| KEGG_NATURAL_KILLER_CELL_MEDIATED_CYTOTOXICITY | -0.6907 | -2.5106 | 0 | 0 | 0 |
| KEGG_B_CELL_RECEPTOR_SIGNALING_PATHWAY | -0.7085 | -2.4488 | 0 | 0 | 0 |
| KEGG_CYTOKINE_CYTOKINE_RECEPTOR_INTERACTION | -0.6516 | -2.4263 | 0 | 0 | 0 |
| KEGG_CHEMOKINE_SIGNALING_PATHWAY | -0.6531 | -2.423 | 0 | 0 | 0 |
| KEGG_JAK_STAT_SIGNALING_PATHWAY | -0.6205 | -2.418 | 0 | 0 | 0 |
| KEGG_T_CELL_RECEPTOR_SIGNALING_PATHWAY | -0.6558 | -2.3717 | 0 | 1.00E-04 | 0.001 |
| KEGG_AUTOIMMUNE_THYROID_DISEASE | -0.8255 | -2.3658 | 0 | 1.00E-04 | 0.001 |
| KEGG_HEMATOPOIETIC_CELL_LINEAGE | -0.7663 | -2.3322 | 0 | 2.00E-04 | 0.002 |
| KEGG_CELL_ADHESION_MOLECULES_CAMS | -0.6993 | -2.3015 | 0 | 2.00E-04 | 0.002 |
| KEGG_LEISHMANIA_INFECTION | -0.7523 | -2.2774 | 0 | 3.00E-04 | 0.003 |
| KEGG_TOLL_LIKE_RECEPTOR_SIGNALING_PATHWAY | -0.597 | -2.2622 | 0 | 3.00E-04 | 0.003 |
| KEGG_FC_EPSILON_RI_SIGNALING_PATHWAY | -0.595 | -2.2521 | 0 | 4.00E-04 | 0.004 |
| KEGG_INTESTINAL_IMMUNE_NETWORK_  FOR_IGA_PRODUCTION | -0.853 | -2.2504 | 0 | 3.00E-04 | 0.004 |
| KEGG_FC_GAMMA_R_MEDIATED_PHAGOCYTOSIS | -0.6006 | -2.2268 | 0 | 3.00E-04 | 0.004 |
| KEGG_VIRAL_MYOCARDITIS | -0.7076 | -2.1716 | 0 | 4.00E-04 | 0.006 |

**Supplementary table 3. Clinicopathological information of patients used for IHC**

| **Case number** | **Age** | **Sex** | **Pathological type** | **Stage** |
| --- | --- | --- | --- | --- |
| **1** | **68** | **female** | **LUAD** | **ⅢA** |
| **2** | **55** | **female** | **LUAD** | **ⅠA** |
| **3** | **47** | **female** | **LUAD** | **ⅠA** |
| **4** | **68** | **female** | **LUAD** | **ⅠA** |
| **5** | **53** | **female** | **LUAD** | **ⅠA** |
