# Supplementary material for: LRMP Associates With Immune Infiltrates and Acts as a Prognostic Biomarker in Lung Adenocarcinoma
Source: Front Mol Biosci. 2021 Nov 26;8:711928. doi: 10.3389/fmolb.2021.711928 (PMC8661541; doi:10.3389/fmolb.2021.711928)

| Gene name | Primers 5’ 3’ |
| --- | --- |
| LRMP | GATCAGCTTCTCCCACGATAGA  ACCGATTTCACAGTGGTTACAG |
| IL16 | ACGAAGCTACTTGACGAAAAGAC  GTTTCAGCAGAACCATTTGCAG |
| KLHL6 | CCAGAAAACTGCGTTGGAATACT  GGTCACGTAAAGGTCATCACTC |
| EVI2B | ACCAACACAATTCAGCGACAC  GTTGTAGGCAAGTGGTTGTCC |
| SASH3 | AAGGTGGGCTCTTTCAAATTCA  CATGCAGGGTCTTAGGCTTGG |
| ARHGAP25 | TCCATCCTTCCTCGTGACAAC  CGACCTGATGAGATTCACACCAA |
| IKZF1 | CATCAGCCCGATGTACCAGC  CCTCGTTGTTGCTCTCGGT |
| CD274 | GCTGCACTAATTGTCTATTGGGA  AATTCGCTTGTAGTCGGCACC |
| GAPDH | GCACCGTCAAGGCTGAGAAC  TGGTGAAGACGCCAGTGGA |

Supplementary data 1. Primers used in this study.

Supplementary data 2. Antibodies used in this study.

| Antibody name | Manufacturer | Dilution |
| --- | --- | --- |
| LRMP | Abcam(ab202418) | IHC:1:100  WB1:1000 |
| p-STAT3 | CST (9145S) | 1;1000 |
| p-PI3K | CST(17366S) | 1:1000 |
| p-AKT | CST（4060S） | 1:1000 |
| p-MEK | CST(9121S) | 1:1000 |
| E-cadherin | CST(3195S) | 1:1000 |
| N-cadherin | CST(13611S) | 1:1000 |
| Slug | CST(9585S) | 1:1000 |
| GAPDH | CST(5174S) | 1:1000 |

Supplementary data 3. Certificate for vector construction.

| **CERTIFICATE OF ANALYSIS** | | | | | | | | |
| --- | --- | --- | --- | --- | --- | --- | --- | --- |
| **Gene Name** | pCMV-Tag2B- IRAG2 | | | | | **Order No.** | G0200963-1 | |
| **Lot No.** | C23510 | | | | | **Cloning Vector** | pCMV-Tag2B | |
| **Cloning Sites** | BamHI(GGATCC)-XhoI(CTCGAG) | | | | | **Insert Size** | 1509bp | |
| **Competence** | Top10 | | | | | **Vector Resistance** | Kan | |
| **QC Results** | | | | | | | | |
| **Test Items** | | **Specifications** | | | | | | **Results** |
| **Insert Sequence** | | Insert sequence results consistent with target | | | | | | Pass |
| **Vector Sequence** | | Flanking sequence consistent with expected | | | | | | Pass |
| **ORF Across Junction** | | Correct and consistent with target | | | | | | N/A |
| **Restriction Digest** | | Expected fragment sizes observed | | | | | | Pass |
| **PCR Amplification** | | Correct without non - specific bands | | | | | | N/A |
| **DNA Quantity/Quality** | | Actual yield (by A 260 ) | | | | | | 5μg/5μg |
|  |  | Concentration (n/a if lyophilized) | | | | | | N/A |
|  |  | Purity (A 260/A280 = 1.8 - 2.0) | | | | | | Pass |
|  |  | # of Tubes | | | | | | 2 |
|  |  | Matrix | | | | | | TE (lyophilized) |
| **Endotoxin Test** | | Verified, <0.1 EU/µg (Endo-Free Preps Only) | | | | | | N/A |
| **Appearance** | | Clear, no visible particles | | | | | | Pass |
| **Label** | | Correct and white | | | | | | Pass |
| **Comments** | | - | | | | | | - |
| **Restriction Digestion Map** | | | | | | | | |
|  | | **1** | **2** | **M** |  | | | |
| 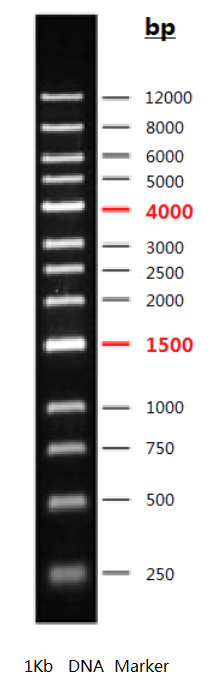 | | 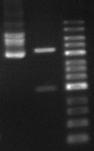 | | | **Lane 1：**Plasmid  **Lane 2**: Plasmid Digested with BamHI--XhoI  **Lane M**: DNA Marker | | | |

Supplementary data 4. Sequencing result of amplified fragment (LRMP).


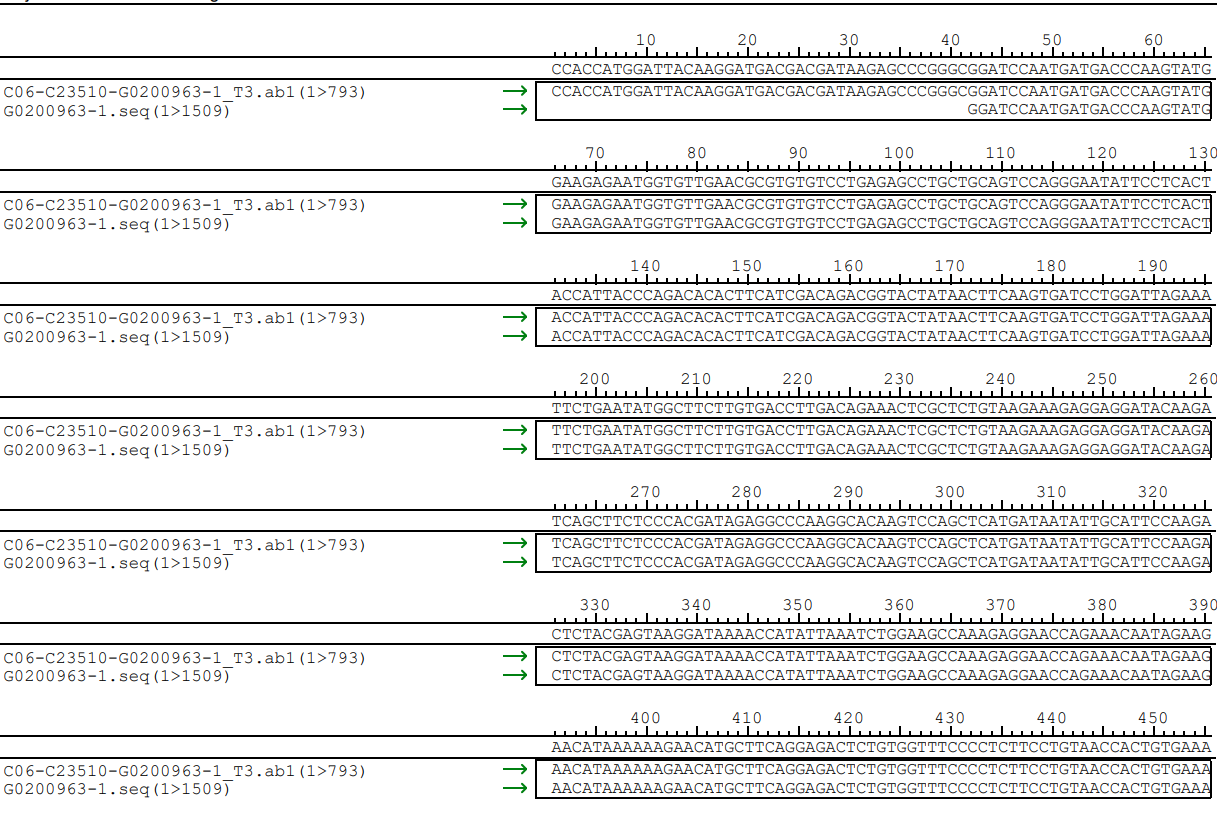

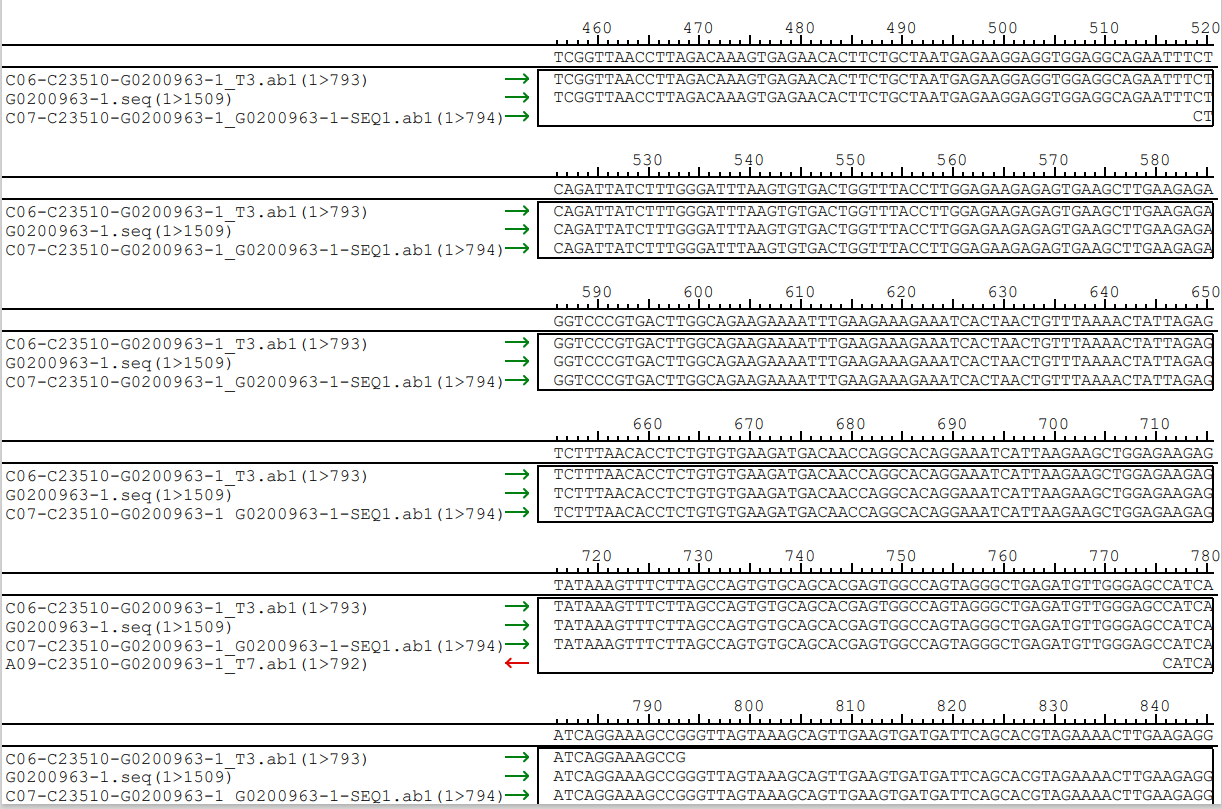

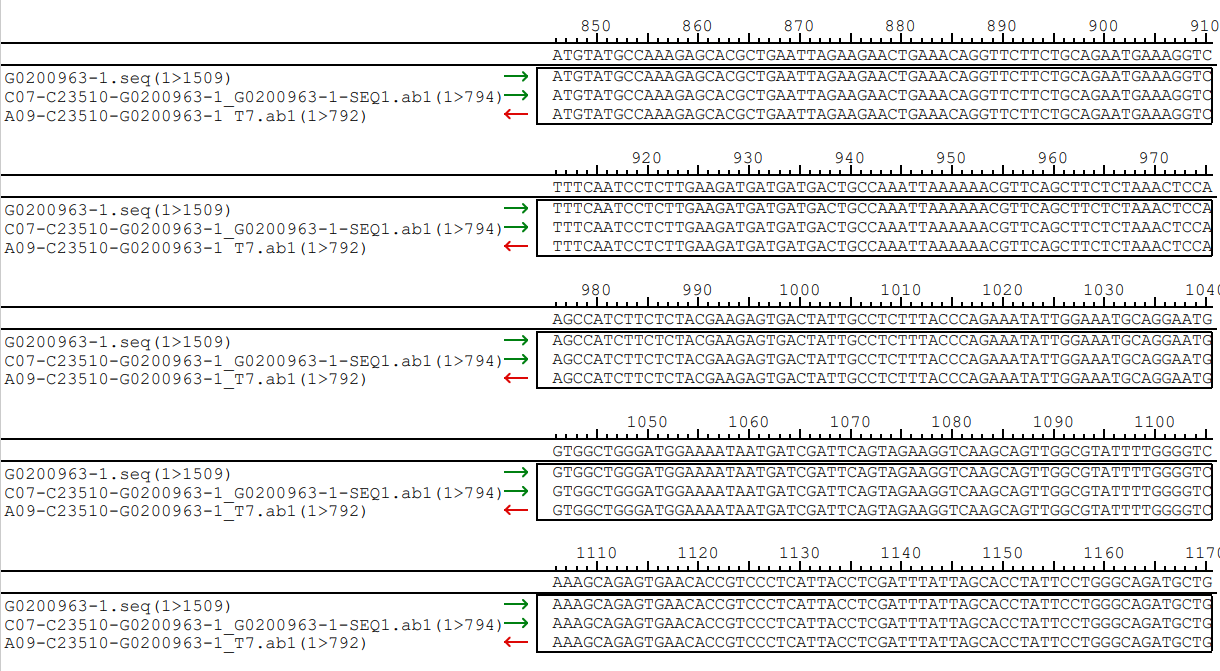

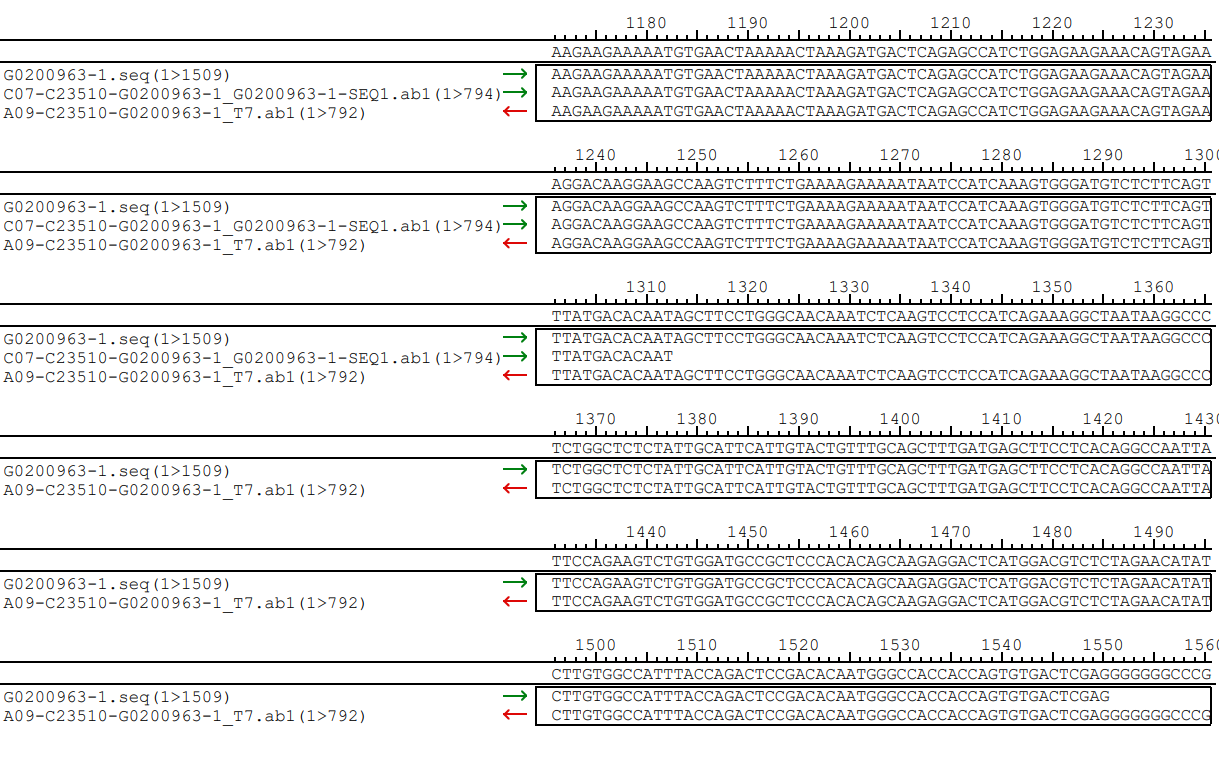

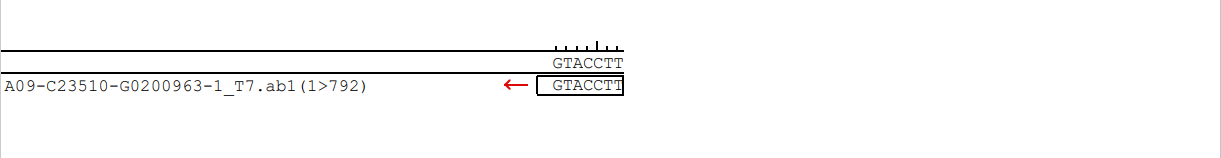

Supplement: Supplementary file 7 [file DataSheet1.docx]
